# PTBP2-Mediated Alternative Splicing of IRF9 Controls Tumor-Associated Monocyte/Macrophage Chemotaxis and Repolarization in Neuroblastoma Progression

**DOI:** 10.34133/research.0033

**Published:** 2023-01-30

**Authors:** Jue Tang, Jing He, Huiqin Guo, Huiran Lin, Meng Li, Tianyou Yang, Hai-Yun Wang, Di Li, Jiabin Liu, Le Li, Huimin Xia, Zhenjian Zhuo, Lei Miao

**Affiliations:** ^1^ Department of Pediatric Surgery, Guangzhou Institute of Pediatrics, Guangdong Provincial Key Laboratory of Research in Structural Birth Defect Disease, Guangzhou Women and Children’s Medical Center, Guangzhou Medical University, Guangzhou 510623, Guangdong, China.; ^2^ School of Medicine, South China University of Technology, Guangzhou, Guangdong 510623, China.; ^3^ Faculty of Medicine, Macau University of Science and Technology, Macau 999078, China.; ^4^ Laboratory Animal Center, School of Chemical Biology and Biotechnology, Peking University Shenzhen Graduate School, Shenzhen 518055, China.

## Abstract

The recurrence and metastasis of children with mediastinal neuroblastoma (NB) are also occurred after surgery, chemotherapy, or radiotherapy. Strategies targeting the tumor microenvironment have been reported to improve survival; however, thorough investigations of monocytes and tumor-associated macrophages (Mϕs) with specialized functions in NB are still lacking. Our data first demonstrated polypyrimidine tract binding protein 2 (PTBP2) as a possible identifier in patients with mediastinal NB screened by proteomic profiling and that PTBP2 predicted good outcomes. Functional studies revealed that PTBP2 in NB cells induced the chemotactic activity and repolarization of tumor-associated monocytes and Mϕs, which, in turn, inhibited NB growth and dissemination. Mechanistically, PTBP2 prevents interferon regulatory factor 9 alternative splicing and upregulates signal transducers and activators of transcription 1 to stimulate C-C motif chemokine ligand 5 (CCL5) and interferon-stimulated gene factor-dependent type I interferon secretion, to induce monocyte/Mϕs chemotaxis, and to sustain monocytes in a proinflammatory phenotype. Our study defined a critical event of PTBP2-induced monocytes/Mϕs in NB progression and revealed that RNA splicing occurred by PTBP2 benefits immune compartmentalization between NB cells and monocytes. This work revealed the pathological and biological role of PTBP2 in NB development and indicates that PTBP2-induced RNA splicing benefits immune compartmentalization and predicted a favorable prognosis in mediastinal NB.

## Introduction

Monocytes exert the ability to derive tumor “conditioned” monocytes and macrophages (Mϕs) to shape the tumor microenvironment (TME) [1]. Tumor-associated Mϕs (TAMs) with a proinflammatory subtype act as a tumor-inhibiting role during cancer progression, a consistent protumor phenotype usually linked to M2 polarization of Mϕs [2,3]. Clinical studies have shown that high circulating monocytes and TAMs are correlated with poor prognosis in several adult solid cancers [4,5]. However, the phenotype of these cells, especially in vivo, is more complicated than that of clear-cut M1- or M2-polarized states [6]. A better understanding of how to balance monocyte fates in antitumoral immunity will be more effective, especially in immunotherapies.

In children with metastatic neuroblastoma (NB), the overall survival (OS) is still low because of unpredictable dissemination and relapse in different stages [7]. Effective therapeutic strategies targeting immune cell can lead to improved survival for these children. NB cells have the ability to reprogram circulating monocytes into an immunosuppressive phenotype on T cells and other cells in the TME [8]. To date, patients with NB are also poorly responsive to immune checkpoint blockade [9]. Although much has been revealed from epigenetic and transcriptomic profiling [10,11], intercellular signaling targeting the TME within NBs remains difficult to characterize. Thorough investigations of monocytes and TAMs in their development and specialized functions in NB cells are still lacking. Therefore, a comprehensive study of monocytes and Mϕs in patients with NB and related mechanisms is needed to educate these cells to a tumor-promoting phenotype.

Mature RNAs are formed by alternative splicing of premature mRNA through removal of introns and union of exons in gene transcripts [12]. Different mRNA splice variants resulting from the spliceosome and trans-acting proteins have been reported to encode different protein isoforms to exert distinct functions [13,14]. This delicate system is frequently disrupted in human diseases, including cancers [15]. Polypyrimidine tract binding protein 1 (PTBP1) (also known as PTB or hnRNP I) and its paralog, PTBP2 (also known as nPTB or brPTB), are characterized as trans-acting splicing repressors and play important roles in mRNA regulation [16]. A previous study reported that PTBP1 downregulates PTBP2 through an alternative splicing event [17]. Moreover, PTBP1 and PTBP2 showed mutually exclusive expression in most tissues, PTBP1 was highly expressed, and PTBP2 remained low, which showed the opposite effect in the brain, such as neuronal differentiation, indicating that PTBP2 regulates neuronal maturation by an alternative splicing program [18–20]. However, the roles of RNA splicing in NB progression remain unclear. Our study first demonstrated PTBP2 to be a possible identifier in patients with mediastinal NB with favorable follow-ups from proteomic characterization. Here, we aimed to determine the expression profile of PTBP2 and to further identify its RNA splicing role in NB progression. Functional and mechanical studies were also conducted to demonstrate the effect of PTBP2 in NB cells on circulating monocytes/Mϕs. These findings indicated that PTBP2-induced RNA splicing benefits immune compartmentalization between cancer cells and myeloid cells and predicted a favorable prognosis in mediastinal NB.

## Results

### PTBP2 predicts favor survival of patients with NB

**Fig. 1.**
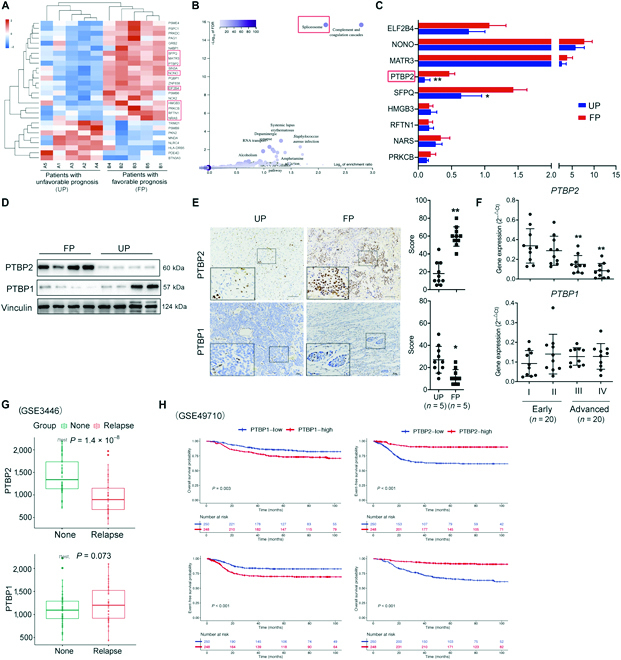
PTBP2 predicts favor survival of patients with NB. (A) Heatmap of substantial altered proteins from the quantitative proteomic profile in 10 NB tumors with UP or FP after chemotherapy (*n* = 5 in each group). Proteins were labeled with TMTs and analyzed. (B) KEGG pathway enrichment analysis of the quantitative proteomic profile. Pathway analysis was performed with the gene set enrichment analysis method, which was based on an empirical permutation test procedure. (C) RT-PCR analysis of cellular alternative splicing process-related genes in NB tumors with UP or FP. The data are presented as the means ± SD. *P < 0.05 and **P < 0.01. (D) Immunoblot analysis showing PTBP1 and PTBP2 expression in NB tumors with UP or FP. Vinculin was used as a loading control. (E) Left: Representative pictures of PTBP1 and PTBP2 IHC staining in NB tumors with UP or FP. Right: The average positive ratio from 3 to 5 fields was counted and identified by symbol and color. *P < 0.05 and **P < 0.01. Scale bars, 100 μm (PTBP2) and 50 μm (PTBP1). (F) The mRNA expression of *PTBP1* and *PTBP2* in different phases (I to IV) of throactic NB samples. **P < 0.01 (G) Bioinformatic analysis of PTBP1 and PTBP2 expression in NBs with or without relapse from the publicly accessible NB database (GSE3446). (H) The OS and event-free survival information of PTBP1 and PTBP2 in publicly accessible NB database (GSE49710).

To understand the underlying mechanism of distinct prognoses among mediastinal NBs, we performed quantitative proteomic analysis of NB samples after chemotherapy. Multiple proteins were substantially altered in patients with FP-NB (Favorable prognosis-neuroblastoma patients) relative to those in patients with UP-NB (Unfavorable prognosis-neuroblastoma patients”) (Fig. 1A and Fig. S2A). Notably, cellular alternative mRNA splicing pathway process-related proteins, such as splicing factor proline and glutamine rich (SFPQ), matrin 3 (MATR3), PTBP2, non-POU domain containing octamer binding (NONO), eukaryotic translation initiation factor 2B subunit delta (EIF2B4), high mobility group box 3 (HMGB3), protein kinase C beta (PRKCB), raftlin, lipid raft linker 1 (RFTN1), and NRAS proto-oncogene, GTPase (NRAS), were significantly upregulated in patients with FP-NB (Fig. 1A). Gene Ontology (GO) and Kyoto Encylclopedia of Genes and Genomes (KEGG) analyses also showed that the alternative splicing (via spliceosome) pathway was obviously enriched (Fig. 1B and Fig. S2B and C). Further mRNA level analysis of differentially expressed proteins involving alternative splicing pathway indicated that *PTBP2* was the most significantly upregulated gene in patients with FP-NB (Fig. 1C). Because PTBP2 and its paralog PTBP1 are both mutually exclusive and critical for neuronal differentiation [18,19], we further detected PTBP1 and PTBP2 expression in 2 subsets. As expected, the Western blot and immunohistochemistry (IHC) staining data showed that more PTBP2 was expressed in FP-NB tumors than in UP-NB tumors, while PTBP1 was abundantly expressed in UP-NB tumors (Fig. 1D and E). Moreover, *PTBP2* expression was also significantly higher in early stage (stages I and II) than in advanced stage (stages III and IV) NB tissues, according to stage classification (Fig. 1F). However, the expression of *PTBP1* was not obviously altered (Fig. 1F). Overall, our data demonstrated that PTBP1- and PTBP2-mediated alternative splicing (AS) events play a certain role in NB progression.

We further determined PTBP1 and PTBP2 expression in children and nerve-related tumors from public microarray datasets. The expression of PTBP2 was lower in NBs with relapse than in those without relapse, while PTBP1 was not obvious (Fig. 1G) [Gene Expression Omnibus (GEO): GSE3446]. In the GSE49710 dataset, the OS and event-free survival (EFS) information of PTBP2 displayed better favorable survival, while PTBP1 showed the opposite effect (Fig. 1H). Analysis of death of disease and progression also showed that PTBP2 had lower expression in NB with death or progression than in live or nonprogressive NB, while PTBP1 showed the opposite expression (Fig. S2D).

In other pediatric tumors, PTBP2 was expressed at low levels in the metastasis and death cohorts of hepatoblastoma, whereas PTBP1 was highly expressed (Fig. S2E; GEO: GSE131329). We further acquired the OS and recurrence-free survival information of PTBP1/PTBP2 in these datasets. High PTBP2 expression was significantly associated with good survival of glioblastoma, rather than nephroblastoma, while high PTBP1 expression predicted unfavorable survival in both glioblastoma and nephroblastoma (Fig. S2F to H). These results suggested that PTBP2 is negatively associated with pediatric tumor progression. In particular, PTBP2 may be a tumor-inhibiting gene in both stage- and prognosis-dependent NB tumors.

### PTBP2 indirectly affects NB progression

**Fig. 2.**
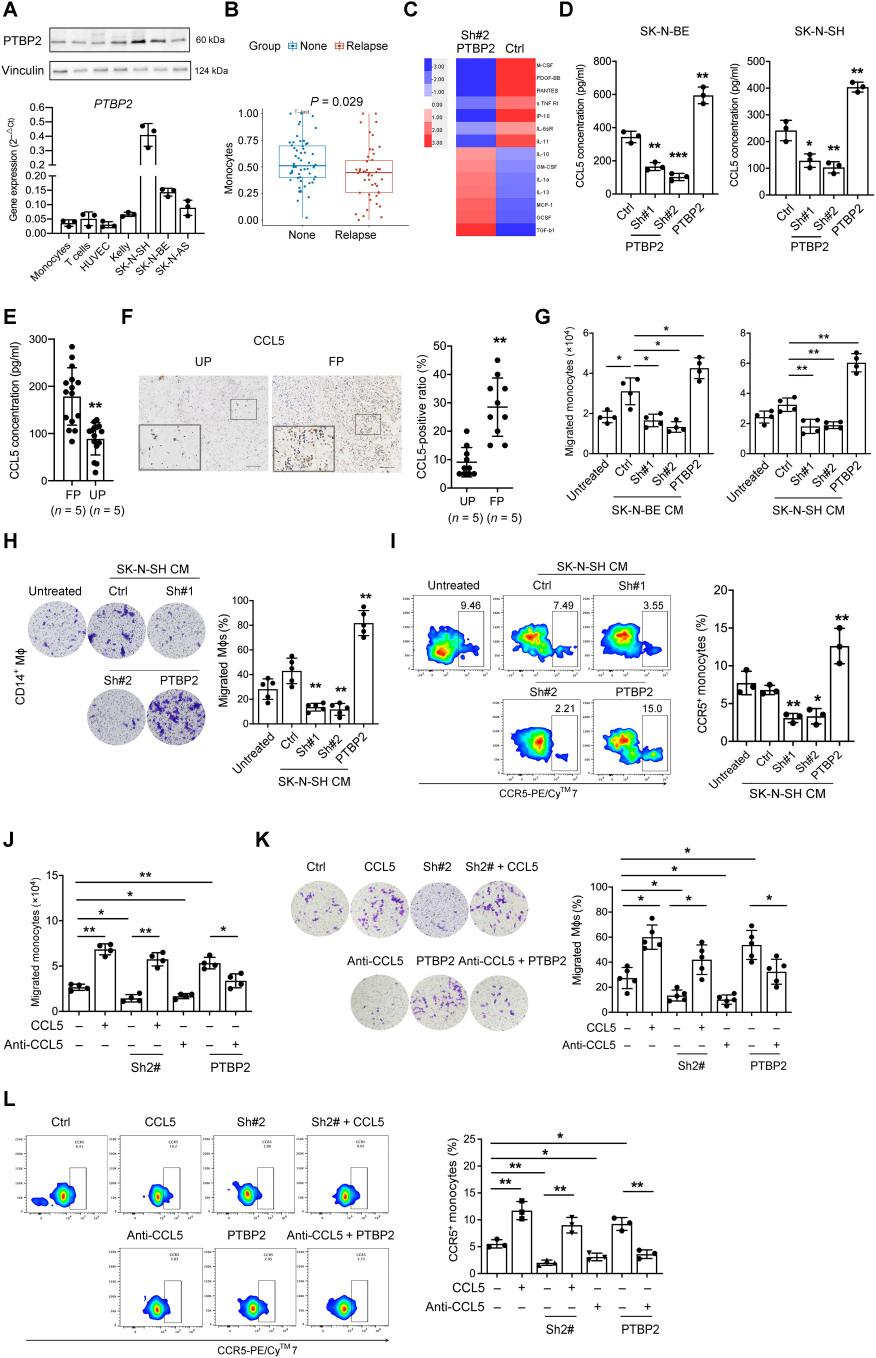
PTBP2-induced chemotactic activity of monocytes and Mϕs through CCL5. (A) Immunoblot and RT-PCR analysis showing PTBP2 expression in NB cell lines, HUVECs, total T cells, and monocytes from patients with NB. (B) Bioinformatic analysis of monocyte infiltration in the NB-TME (GSE3446). (C) Heatmap of differentially expressed cytokines from inflammatory cytokine arrays (AAH-CYT-G5-4) exposed to PTBP2 knockingdown. (D and E) ELISA of CCL5 concentration in the supernatant of SK-N-BE and SK-N-SH cells treated with PTBP2, as well as NB tumors. (F) Left: Representative pictures of CCL5 IHC staining in FP-NB and UP-NB tumors. Right: The average positive ratio from 3 to 5 fields was determined and identified by symbol and color. Scale bars, 100 μm. (G and J) The chemotaxis of CD14^+^ monocytes treated as indicated was analyzed by Transwell assays. The number of monocytes migrating to the bottom chamber was counted and statically analyzed. (H and K) The chemotaxis of CD14^+^ monocyte-derived Mϕs treated as indicated was analyzed by Transwell assays. (I and L) Left: Representative flow cytometry plots of CCR5^+^ monocytes treated as indicated. The percentages are shown and analyzed in the right panel. The data are presented as the means ± SD (*n* = 3). *P < 0.05 and **P < 0.01. Ctrl in (D) and (G) to (L) stands for the average of undistinguishable controls of scrambled sequence (for Sh-PTBP2) and empty vector (for PTBP2 overexpression).

Cellular expression of PTBP2 was detected in 4 NB cell lines, human umbilical cord endothelial cells (HUVECs), pan T cells, and monocytes from patients with NB. High PTBP2 expression was observed in NB cells, especially in SK-N-BE and SK-N-SH cells (Fig. 2A). To elucidate the effects of PTBP2 on NB cell behaviors, we knocked down or overexpressed PTBP2 in SK-N-BE and SK-N-SH cells. The efficiency of PTBP2 was validated via immunoblotting and reverse transcription polymerase chain reaction (RT-PCR; Fig. S3A). Unexpectedly, PTBP2 expression did not significantly affect the viability of NB cells (Fig. S3B and C) but did slightly affect the migration ability of NB cells (Fig. S3D), suggesting that PTBP2 might affect NB progression through other cells indirectly in TME.

### PTBP2-induced chemotactic activity of monocytes and Mϕs through CCL5

Immune cell infiltration analysis was used to detect immune-related differences in the NB-TME (GSE3446). Many myeloid cells and lymphocytes significantly accumulated in relapsed tissue, and only monocytes and T_H_2 helper cells were reduced in NBs with relapse (Fig. 2B and Fig. S4). We then compared supernatants from PTBP2-altered NB cells through inflammatory cytokine array measurement (AAH-CYT-G5-4). Among the numerous cytokines that were changed (Table S3), RANTES (CCL5) drew our attention (Fig. 2C and Fig. S3E). CCL5 and its receptor CCR5 play a role in the inflammatory response by directing cells (monocytes, TAMs, and regulatory T cells) to sites of inflammation [21]. We further verified that the concentration and mRNA levels of CCL5 were increased after overexpression and decreased by PTBP2 knockingdown in NB cells, respectively (Fig. 2D and Fig. S3F). As expected, the concentration and expression of CCL5 in favorable prognosis tumors were significantly higher than that in unfavorable prognosis tumors (Fig. 2E and F).

NB-CM (conditional medium) from PTBP2-altered NB cell lines was added to the bottom of the Transwell inserts. Interestingly, NB-CMs from SK-N-SH and SK-N-BE cells had chemotactic activity for monocytes and monocyte-derived Mϕs (Fig. 2G and H), which was hampered by PTBP2 knockingdown or promoted by PTBP2 overexpression. Concomitantly, PTBP2 knockingdown decreased CD14^+^CCR5^+^ monocytes, while PTBP2 overexpression increased them (Fig. 2I and Fig. S3G).

Functional blocking and rescue assays further demonstrated the role of CCL5 in PTBP2-altered NB cells. The CCL5-neutralizing antibody significantly inhibited the chemotactic activity of both monocytes and Mϕs, as well as CCR5 expression. Moreover, PTBP2-induced migration and CCR5 expression were further inhibited by CCL5 neutralization. Conversely, human CCL5 not only increased the chemotactic activity of both monocytes and Mϕs, as well as CCR5 expression, but also rescued the decreased level of PTBP2-knockingdown induced CCL5 (Fig. 2J to L). These results indicated that the PTBP2-induced chemotactic activity of monocytes and Mϕs depends on the CCL5/CCR5 axis.

### Monocytes/Mϕs exposed to PTBP2-treated CM are responsible for NB cell proliferation and migration

**Fig. 3.**
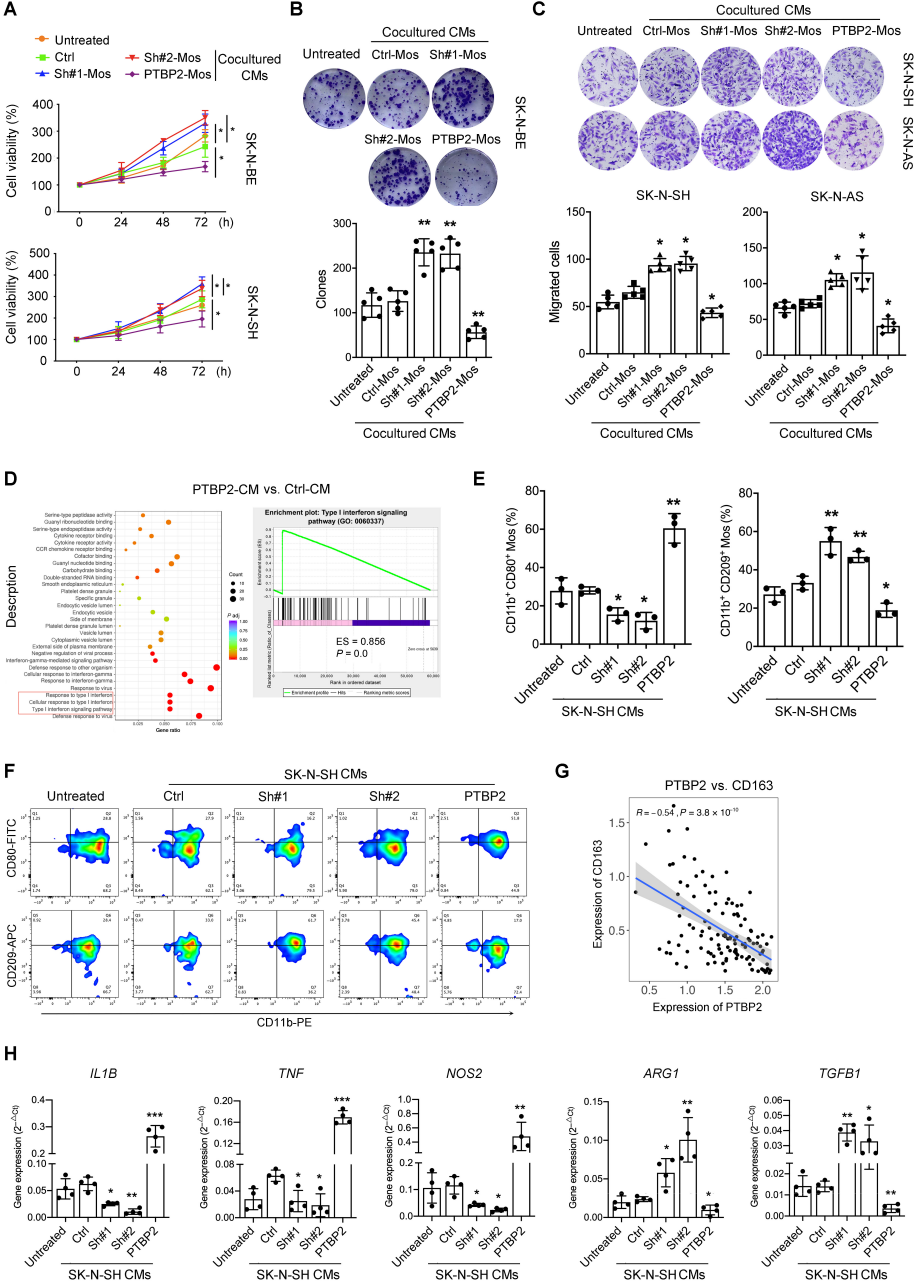
Monocytes (Mos)/Mϕs educated by PTBP2-treated NB cells inhibits NB cell proliferation and migration. (A) The proliferation rate of human SK-N-BE and SK-N-SH cells exposed to co-CMs treated as indicated was assessed by CCK-8 assay. (B) Colony formation assay and statistical analysis of SK-N-BE cells subjected to co-CM treatment as indicated. (C) Transwell assay and statistical analysis of NB cells subjected to co-CM treatment as indicated. (D) KEGG pathway enrichment analysis of RNA-seq expression profiling in Mϕs with PTBP2 knockingdown. Pathway analysis was also performed with the gene set enrichment analysis method, which was based on an empirical permutation test procedure. (E and F) Representative flow cytometry plots of CD11b^+^CD80^+^ and CD11b^+^CD209^+^ monocytes treated as indicated. The percentages are shown and analyzed in (E). (G) Correlation analysis showing the expression of PTBP2 and CD163 in the online microarray dataset (GSE3446). (H) RT-PCR analysis of *IL1B*, *TNF*, *NOS2*, *Arg1*, and *TGFB* expression in Mϕs treated as indicated. The data in (A) to (C), (E), and (H) are representative of 3 independent experiments and presented as the means ± SD; *P < 0.05, **P < 0.01, and ***P < 0.001. Ctrl represents the average of undistinguishable controls of scrambled sequences (for Sh-PTBP2) and the empty vector (for PTBP2 overexpression).

Cocultured CM (co-CM) was obtained from coculture system of PTBP2-treated NB cells and monocytes and then added to NB cells. Although co-CM from PTBP2 overexpression group seldomly affect the cell cycle transition of SK-N-BE and SK-N-SH cells (Fig. S5B), CCK-8 and clone assays revealed that the proliferation of NB cells was significantly inhibited by co-CM from PTBP2-overexpressing group, while promoted by PTBP2 knockingdown co-CM, respectively (Fig. 3A and B). Furthermore, apoptotic analysis revealing that co-CM from PTBP2-overexpressed group significantly induced NB cell apoptosis, while PTBP2-knockingdown-derived co-CMs showed a tendency but not significant effect to decrease apoptosis of NB cells (Fig. S5A). Interestingly, co-CM from the PTBP2 overexpression group obviously inhibited the migration of NB cells, while the PTBP2 knockingdown group showed the opposite effect (Fig. 3C), which is in accordance with our finding from NB tissues that the tumor-inhibiting effect of PTBP2 was dependent on the chemotactic activity of monocytes and Mϕs in the TME. Therefore, our data demonstrated the indirect effects of PTBP2 on NB cells, through affecting migrated monocytes/Mϕs, which were contributed by the proliferate inhibition, proapoptotic, and migrate activities.

However, neither CCL5- nor anti-CCL5-neutralizing antibody affected NB cell migration and clone abilities induced by the PTBP2-altered NB cell–monocyte system (Fig. S5C and D), indicating that the tumor-inhibiting effect of PTBP2-induced monocytes/Mϕs was not determined by the CCL5/CCR5 axis.

RNA sequencing (RNA-seq) was performed in Mϕs subjected to NB-CMs from PTBP2 overexpression and Ctrl. In total, 505 downregulated and 473 upregulated genes were screened in the PTBP2 knockingdown group compared with the Ctrl (Fig. S5E). GO analysis revealed that the IFN-I signaling pathway was significantly enriched (Fig. 3D). Furthermore, NB-CMs of PTBP2 knockingdown decreased CD11b^+^CD80^+^ monocytes and increased the CD11b^+^CD209^+^ subset. Conversely, NB-CMs of PTBP2 overexpression exerted the opposite effects (Fig. 3E and F). CD163, another anti-inflammatory marker of monocytes/Mϕs, was also detected to have a significant negative correlation (*R* = −0.54) with PTBP2 expression in the online microarray dataset (GEO: GSE3446) (Fig. 3G). RT-PCR analysis also showed that inflammatory cytokines such as *IL1B*, *TNF*, and *NOS2* were downregulated or upregulated after exposure to NB-CM with PTBP2 knockingdown or overexpression, which conversely increased or decreased *Arg1* and *TGFB* expression, respectively (Fig. 3H). Moreover, both CCL5 and anti-CCL5 antibodies hardly reversed the phenotype of monocytes altered by PTBP2 (Fig. S5F and G). Overall, our results show that the switch of monocytes to the inflammatory subset and IFN-I signaling activation by PTBP2-overexpressing NB cells, which were responsible for the tumor-inhibiting effect, was independent of the CCL5/CCR5 axis.

### PTBP2 knockingdown induces alternative splicing of IRF9 within the exon 6-7-8 region

**Fig. 4.**
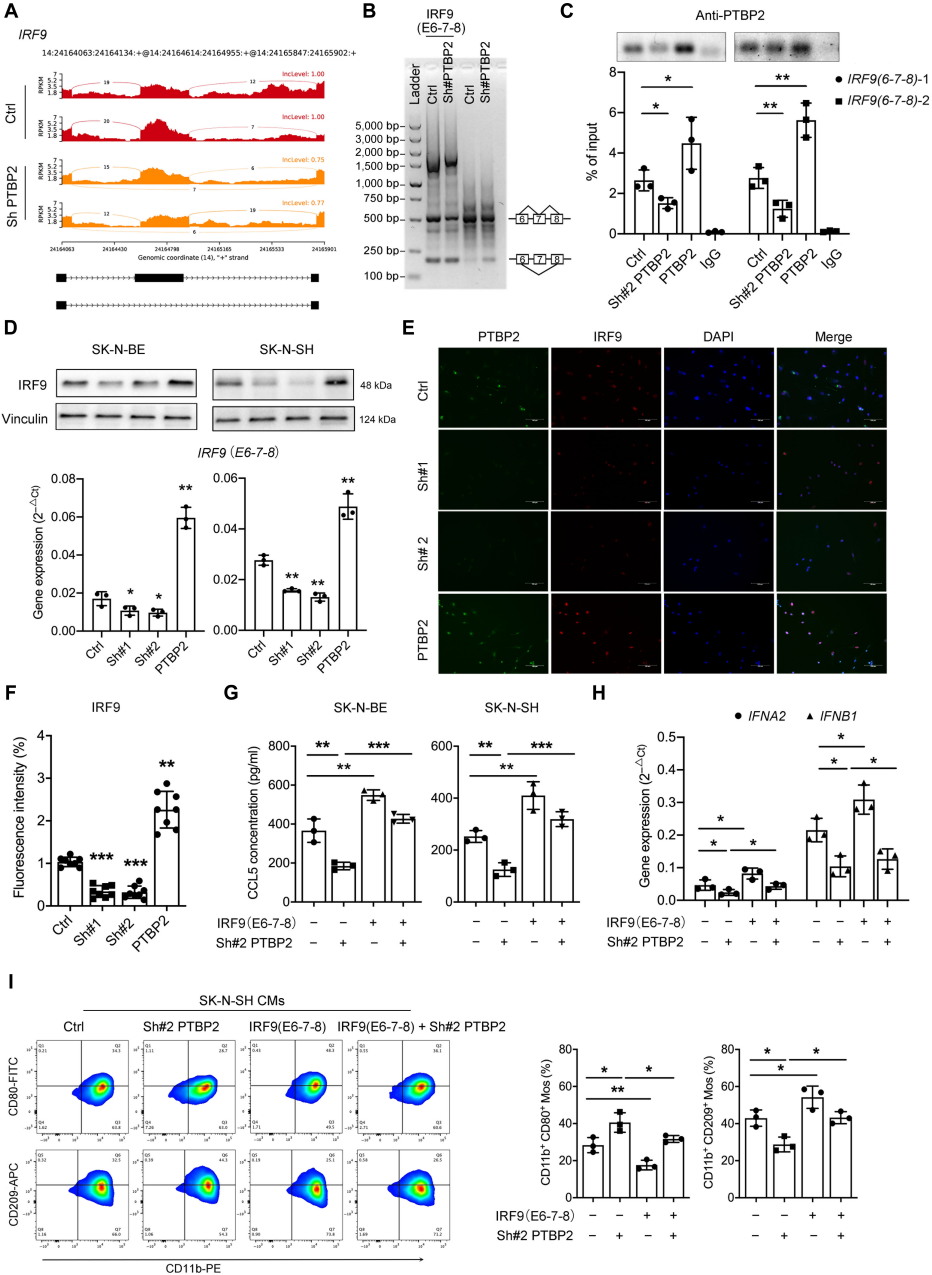
PTBP2-induced alternative splicing of IRF9 affects CCL5 and IFNα/β expression. (A) Alternative splicing event of IRF9 in exon skipping was from RNA-seq reads in the PTBP2 knockingdown and control groups. (B) RT-PCR of *IRF9* (exon 6-7-8) splicing in SK-N-SH cells treated as indicated. The products were further separated by DNA gels. (C) The direct binding between PTBP2 and the exon 6-7-8 region of *IRF9* was measured by CLIP-PCR assay, The cDNA band in each group was shown in the top panel. IRF9(6-7-8)-1 and IRF9(6-7-8)-2 refer to 2 primers targeting at 2 regions of IRF9(6-7-8). (D) RT-PCR and immunoblot analysis showing *IRF9* expression after treatment, as indicated. (E and F) Representative immunofluorescent staining pictures of PTBP2 (green) and IRF9 (red) in SK-N-SH cells. DAPI (blue) stained for nuclei. Scale bars, 100 μm. The fluorescence intensity of IRF9 was statistically analyzed in the same manner as that in (F). (G) CCL5 concentration in the supernatant of SK-N-BE and SK-N-SH cells treated as indicated was measured by ELISA. (H) RT-PCR analysis of *IFNA2* and *IFNB1* expression after treatment as indicated. (I) Left panel: Representative flow cytometry plots of CD11b^+^CD80^+^ and CD11b^+^CD209^+^ monocytes/Mϕs treated as indicated. Right panel: Quantification of percentage in each group. The data in (C) to (I) are representative of 3 independent experiments and presented as the means ± SD. *P < 0.05, **P < 0.01, and ***P < 0.001. Ctrl represents the average of undistinguishable controls of scrambled sequences (for Sh-PTBP2) and the empty vector (for PTBP2 overexpression). IgG, immunoglobulin G.

Using RNA-seq, we next characterized the molecular signaling pathways regulated by PTBP2. Multiple genes and pathways were enriched in PTBP2-knockingdown or PTBP2-overexpression NB cells (Fig. S6A and B). PTBP2 is known as a splicing factor that initiates a series of splicing programs, especially for neuronal maturation and survival [22]. Therefore, we targeted alternative splicing events that occurred in response to PTBP2 alteration from RNA-seq. Among them, IFN regulatory factor 9 (IRF9) was identified as a candidate splicing event after our analysis for its crucial role in amplifying the early dynamics of IFN-mediated signal transduction [23], which is consistent with our above finding that PTBP2-altered NB cells activated the IFN-I signaling pathway of Mϕs. Moreover, the alternative splicing events of IRF9 by PTBP2 knockingdown started from exon 6 to exon 8, which included exon skipping (Chr14: 24164613-24164955, lncLevel difference: 0.242) and alternative 5′ splice sites (Chr14: 24164062-24164955, lncLevel difference: 0.32) (Fig. 4A and Fig. S6C). We then constructed the exon 6-7-8 region of the IRF9 plasmid and transfected it into NB cells before PTBP2 knockingdown. Our results demonstrated that PTBP2 knockingdown significantly reduced the exon 6-7-8 expression of IRF9 [530 base pairs (bp)] compared with that of the control, while the skipping exon 6-8 expression of IRF9 (188 bp) was increased under normal and exon 6-7-8-overexpressing conditions (Fig. 4B), indicating that PTBP2 knockdown effectively accelerated the exon 6-7-8 region of IRF9 spliced into exon 6-8. Finally, cross-linking immunoprecipitation (CLIP) assays were conducted to determine whether PTBP2 directly binds to the exon 6-7-8 region of IRF9 (Fig. 4C). Using a PTBP2 antibody, we were able to isolate RNAs binding to PTBP2 in each group with or without exon 6-7-8 overexpression. As expected, exons 6-7-8 of IRF9 were immunoprecipitated by PTBP2, and the association was promoted under PTBP2 overexpression conditions and inhibited by PTBP2 knockingdown. This confirms the capacity of PTBP2 to bind to the exon 6-7-8 region of IRF9. This finding reveals a pivotal role for PTBP2 editing activity in IRF9 alternative splicing and possible downstream biogenesis.

### PTBP2-induced alternative splicing of IRF9 affects CCL5 and IFNα/β expression

Prompted by the above findings, we further investigated whether alternative splicing of IRF9 has functions in NB cells. First, PTBP2 knockingdown decreased both the mRNA and the protein levels of IRF9, which was rescued by exon 6-7-8 overexpression, while IRF9 (exon 6-7-8)-overexpressing NB cells had higher mRNA and protein levels of IRF9 than the control cells (Fig. S6D and E), indicating that the exon 6-7-8 region plays a key role in IRF9 transcription and translation. RT-PCR, Western blot, and immunofluorescence (IF) staining confirmed that PTBP2 knockdown or overexpression concomitantly decreased or increased the mRNA and protein levels of IRF9 (Fig. 4D to F). Moreover, IRF9 (exon 6-7-8) overexpression increased the CCL5 concentration but was inhibited by PTBP2 knockingdown in NB cells (Fig. 4G). We also took advantage of small interfering RNAs (siRNAs) targeting IRF9 (Fig. S6F) and found that IRF9 downregulation not only decreased the CCL5 concentration but also inhibited PTBP2-induced CCL5 secretion (Fig. S6G). Considering that IRF9 is best characterized as a transcription factor that mediates [as part of IFN-stimulated gene factor 3 (ISGF3)] the IFN-I response by regulating the downstream expression of ISGs and is needed for efficient transcription of diverse IFNα/β genes [23], we were interested in whether IRF9 regulated the IFN-I signaling pathway responsible for the downstream effects on monocytes/Mϕs. Our data showed that IRF9 (exon 6-7-8) overexpression not only increased *IFNA2* and *IFNB1* expression in NB cells and CD11b^+^CD80^+^ monocytes but also rescued PTBP2-knockingdown-induced inhibition of *IFN* expression and CD11b^+^CD80^+^ monocytes, while CD11b^+^CD209^+^ monocytes showed the reverse tendency (Fig. 4H and I). Conversely, lower IRF9 expression not only decreased *IFNA2* and *IFNB1* expression and CD11b^+^CD80^+^ monocytes but also inhibited PTBP2-induced *IFN* expression and CD11b^+^CD80^+^ monocytes (Fig. S6H and I). Collectively, these data demonstrated that PTBP2-induced alternative splicing of IRF9 accelerated CCL5 and IFNα/β secretion, which plays key roles in monocyte chemotaxis and IFN-I responses.

### PTBP2 activates the ISGF3 complex and induces IFNα/β through upregulated STAT1

**Fig. 5.**
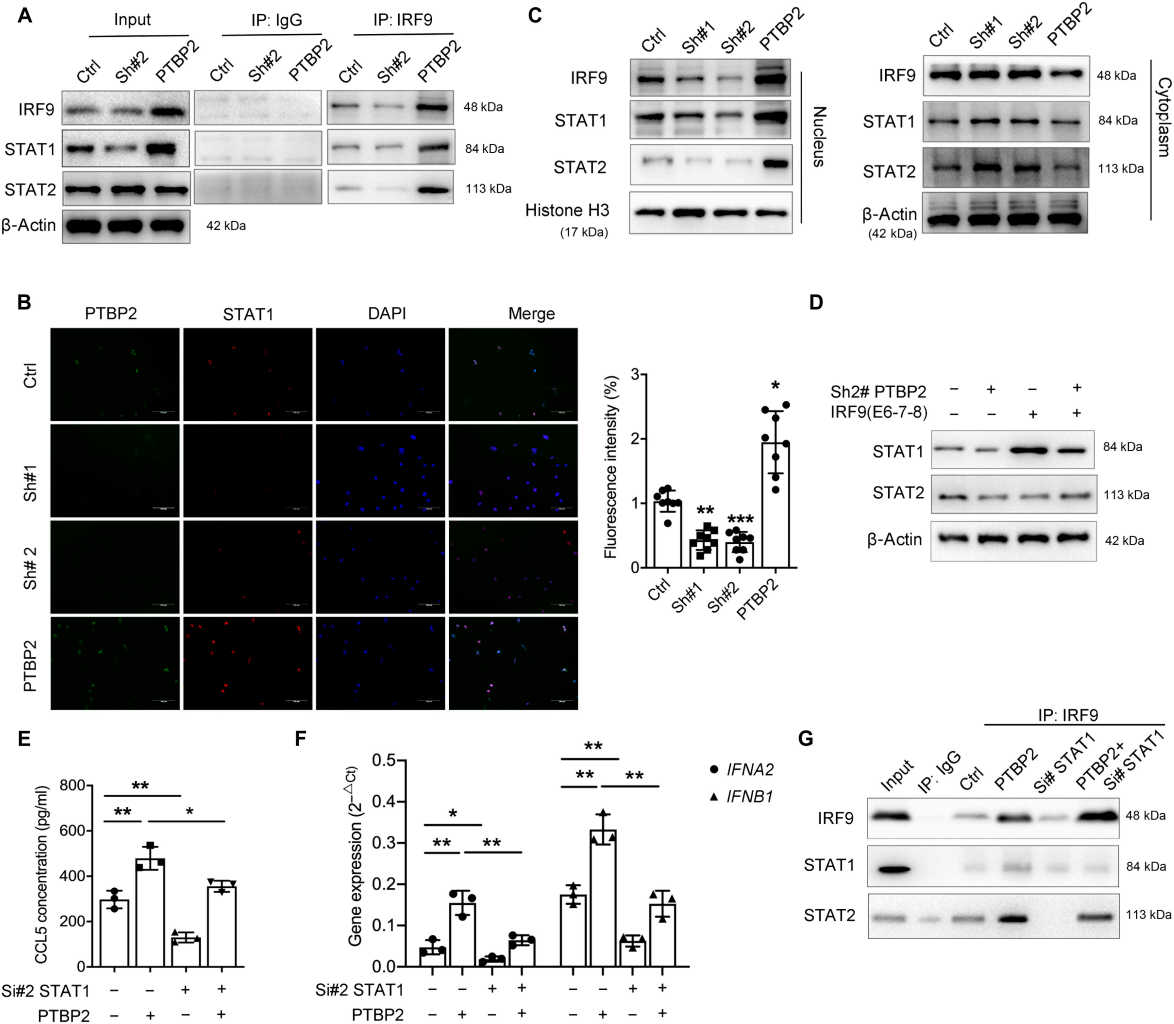
PTBP2-induced IFNs expression is mediated by STAT1 upregulation and ISGF3 activation. (A) Co-IP assays for SK-N-SH with PTBP2 alteration were performed to verify the interaction among IRF9, STAT1, and STAT2. (B) Left: Representative immunofluorescent staining pictures of STAT1 (red) and PTBP2 (green) in SK-N-SH cells. DAPI (blue) stained for nuclei. Scale bars, 100 μm. Right: The fluorescence intensity of STAT1. (C) Immunoblot analysis of IRF9, STAT1, and STAT2 in the cytoplasm and nucleus of SK-N-SH cells with PTBP2 alteration. β-Actin and histone H3 were used as loading controls separately in the cell cytoplasm and nucleus, respectively. (D) Immunoblot analysis of STAT1 and STAT2 in SK-N-SH with PTBP2 alteration. β-Actin was used as a loading control. (E) ELISA for CCL5 concentration treated as indicated. (F) RT-PCR analysis of *IFNA2* and *IFNB1* expression after treatment as indicated. (G) Co-IP assays for SK-N-SH cells treated as indicated were performed to verify the interaction among IRF9, STAT1, and STAT2. The data in (A) to (G) are representative of 3 independent experiments and presented as the means ± SD. *P < 0.05, **P < 0.01, and ***P < 0.001. Ctrl represents the average of undistinguishable controls of scrambled sequences (for Sh-PTBP2) and the empty vector (for PTBP2 overexpression).

Activated signal transducers and activators of transcription 1 (STAT1) in conjunction with IRF9 and STAT2 forms an IFN-activated transcription factor, ISGF3, which is a heterocomplex involved in IFNα/β gene induction. Coimmunoprecipitation (co-IP) studies demonstrated clear associations among IRF9, STAT1, and STAT2 in control NB cells. PTBP2 loss led to a weaker association in the ISGF3 complex, while PTBP2 overexpression showed the reverse effect (Fig. 5A). Interestingly, stable knockingdown or overexpression of PTBP2 also decreased or increased total STAT1 but had no effects on total STAT2 expression; IF staining showed that knockingdown or overexpression of PTBP2 reduced or increased STAT1 (Fig. 5B). In addition, stable knockingdown or overexpression of PTBP2 led to an decrease or increase in nuclear levels of IRF9, STAT1, and STAT2 but slightly affected the cytoplasmic levels of IRF9 and STAT1 in NB cells (Fig. 5C), indicating that PTBP2 alteration has a positive correlation with IRF9 and STAT1 expression, as well as ISGF3 formation. Moreover, IRF9 (exon 6-7-8) overexpression also significantly elevated total STAT1 and rescued PTBP2-knockingdown-induced downregulation of STAT1 (Fig. 5D). Conversely, IRF9 knockingdown not only downregulated total STAT1 but also inhibited PTBP2-induced STAT1 (Fig. S7A), which demonstrated that PTBP2-induced alternative splicing of IRF9 was responsible for total STAT1 expression and ISGF3 complex formation. siRNAs targeting STAT1 and STAT2 were further applied (Fig. S7B and C). We observed that PTBP2-induced CCL5 concentration, ISGF3 complex, and *IFNA2* and *IFNB1* expression were all inhibited by both STAT1 and STAT2 siRNAs (Fig. 5E to G and Fig. S7D to F). The above results demonstrated that the PTBP2/IRF9/STAT1 axis plays a central role in ISGF3 formation to induce a downstream immunoregulatory transcriptional program.

### STAT1 binds to the promoter region of CCL5 and regulates its transcriptional activity

**Fig. 6.**
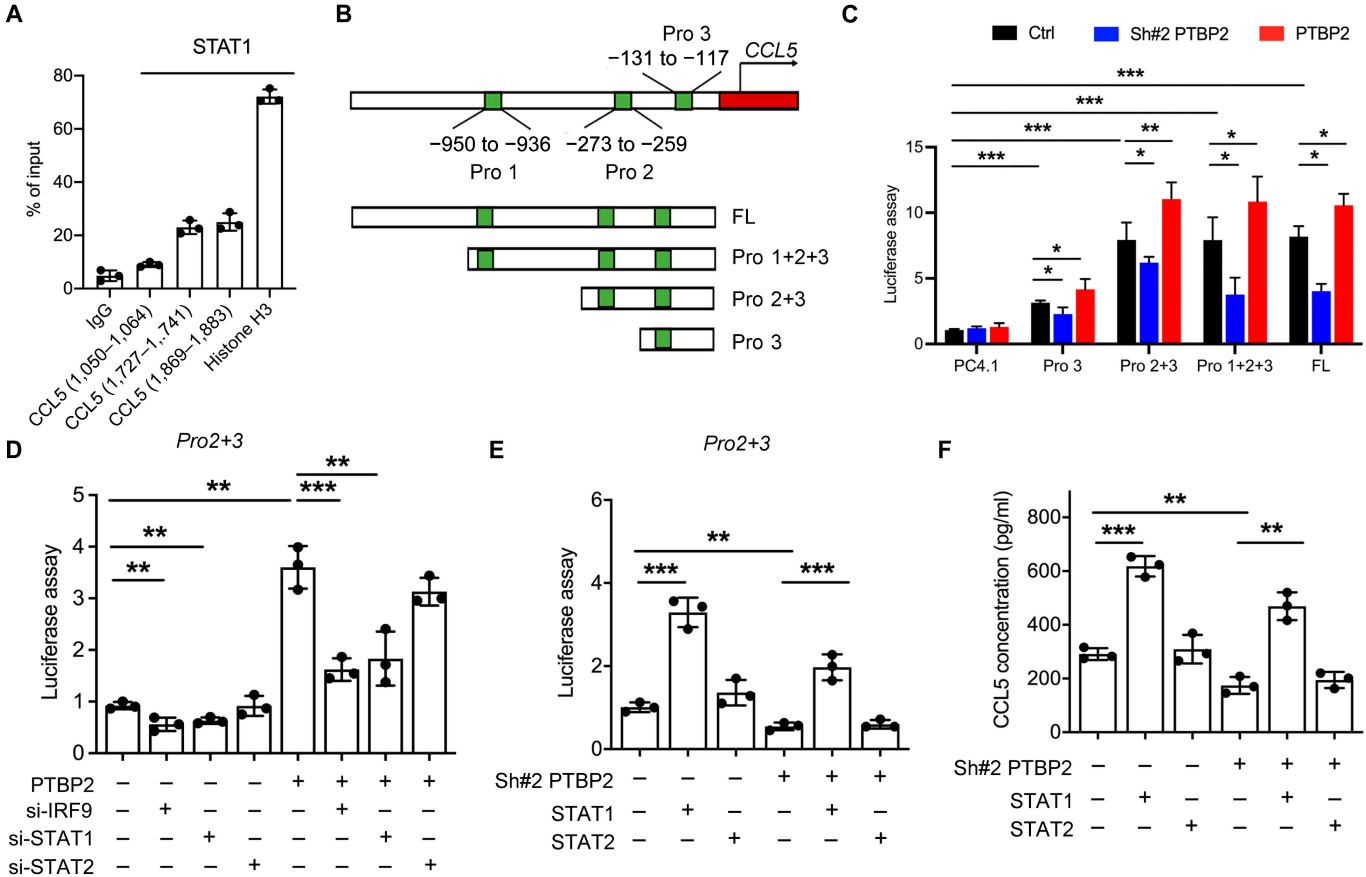
PTBP2-induced CCL5 is transcriptionally regulated by STAT1 and ISGF. (A) ChIP assays showing that the CCL5 promoter contains several STAT1-binding regions. (B) Diagram of possible promoter binding regions of CCL5 by STAT1 and corresponding plasmid construction. (C) Transcriptional activity of CCL5 in SK-N-SH cells transfected with 5 plasmids containing full-length Pro 1+2+3, Pro 2+3, and Pro 3 evaluated by luciferase assays. (D and E) Transcriptional activities of CCL5 in SK-N-SH cells transfected with siRNAs and STAT1- and STAT2-overexpressing plasmids evaluated by luciferase assays. (F) ELISA for CCL5 concentration in SK-N-SH cells treated as indicated. The data in (A) and (C) to (F) are representative of 3 independent experiments and presented as the means ± SD. *P < 0.05, **P < 0.01, and ***P < 0.001. Ctrl represents the average of undistinguishable controls of NC (siRNAs), scrambled sequences (for Sh-PTBP2), and the empty vector (for PTBP2 overexpression).

To ascertain whether the CCL5 gene is a direct target of STAT1, we screened 3 potential STAT1-binding sites in the human CCL5 promoter region (<2,000-bp upstream) through JASPAR database (version 2020) predictions; these sites were mainly located at −131 to −117 bp (Pro 3), −273 to −259 bp (Pro 2), and −950 to −936 bp (Pro 1) (Fig. S8A). Chromatin immunoprecipitation (ChIP) assays showed that STAT1 has a strong affinity for the promoter region of CCL5 at Pro 3 and Pro 2 (Fig. 6A). We constructed 4 plasmids containing full-length Pro 1 + Pro 2 + Pro 3, Pro 2 + Pro 3, and Pro 3 (Fig. 6B) and then separately transfected them into NB cells before PTBP2 treatment. A luciferase assay indicated that the promoter activity of all the groups was reduced after PTBP2 knockingdown but increased after PTBP2 overexpression (Fig. 6C). Moreover, cells transfecting Pro 2 + Pro 3, Pro 1 + Pro 2 + Pro 3, and full length exerted higher promoter activities than Pro 3 (Fig. 6C), confirming that the promoter region of CCL5 contained the predicted STAT1-binding motif located at Pro 2 + Pro 3, which mainly contributed to CCL5 transcription. On the basis of Pro 2 + Pro 3 overexpression conditions, siRNAs targeting IRF9 and STAT1 showed a reduction in promoter activities in both control and PTBP2-transfected SK-N-SH cells, rather than targeting STAT2 (Fig. 6D). However, both the inhibited promoter activity and the decreased CCL5 secretion induced by PTBP2 downregulation were rescued by STAT1 overexpression but were hardly affected by STAT2 overexpression (Fig. 6E and F). Concomitantly, IRF9 (exon 6-7-8) overexpression also exerted the same tendency as STAT1 (Fig. S8B). Overall, our study revealed that STAT1 is a central transcription factor that binds with the −131- to −117-bp and −273- to −259-bp regions of the CCL5 promoter and activates its transcription in NB cells.

### PTBP2 restricts tumor growth by reeducating monocytes in vivo

**Fig. 7.**
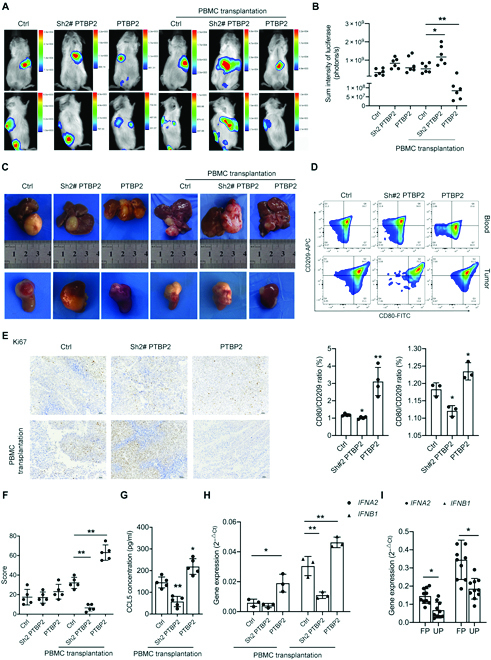
Effects of PTBP2 on tumor growth and metastases in vivo. (A) SK-N-SH-Luc cells with PTBP2 alteration were injected into NDG mice through the tail vein and then subjected to PBMCs. The volumes and sites of metastases were recorded by bioluminescence detection in the front and lateral position of each mouse. (B) Quantification of light emission detected by bioluminescence. Representative images are shown after PBMCs were engrafted. The data are presented as the means ± SD (*n* = 6). *P < 0.05 and **P < 0.01. (C) Visual examination of liver and kidney metastases of mice treated as indicated. (D) Representative flow cytometric histogram of human monocytes stained with CD80 and CD209 in PBMCs and tumors isolated from mice. (E) Representative pictures of Ki67 staining in NBs treated as indicated. Scale bars, 50 μm. The average positive ratio from 3 to 5 fields was analyzed by symbol and color and is shown in (F). (G) ELISA for CCL5 concentration in PBMC-transplanted tumors isolated from mice. (H and I) RT-PCR analysis of *IFNA2* and *IFNB1* expression in PBMC-transplanted tumors isolated from mice and human NB tumors and unfavorable prognosis or favorable prognosis group was extended to 10 samples. *P < 0.05 and **P < 0.01. Ctrl represents a representative sample of undistinguishable controls of scrambled sequences (for Sh2-PTBP2) and the empty vector (for PTBP2 overexpression).

To verify our in vitro data in an in vivo system, we further established a metastatic NB model by injecting stable SK-N-SH cell lines with PTBP2 overexpression or knockdown in the vein (Fig. S9A). Mice were subjected to human peripheral blood mononuclear cells (PBMC) transplantation. First, human CD45^+^ PBMCs were successfully observed in the metastatic sites (6.12%) and blood (8.34%) of mice after transplantation (Fig. S9B). No difference in survival rate was found among these groups (Fig. S9C). In vivo bioluminescence imaging represented that PTBP2 overexpression markedly inhibited the metastatic tumor size, while depletion of PTBP2 expanded the metastatic tumor size exposed to human PBMC transplantation, while no obvious difference was observed among groups without human PBMCs (Fig. 7A and B). Accordingly, a substantially increase in the development of liver and kidney node metastases was observed with the depletion of PTBP2 with human PBMC transplantation. In contrast, PTBP2 overexpression in human PBMCs decreased liver and kidney node metastases (Fig. 7C). Furthermore, compared with that in the control group with PBMC transplantation, PTBP2 overexpression showed a higher ratio of human CD11b^+^CD80^+^ to CD11b^+^CD209^+^ monocytes/Mϕs in blood and tissue, which was lower in PTBP2 knockingdown (Fig. 7D). Ki67 staining of tumor sections indicated that knockingdown or overexpression of PTBP2 with human PBMCs promoted or suppressed the proliferation of tumor cells in mice, respectively (Fig. 7E and F). In addition, PTBP2-induced human CCL5 concentration, as well as *IFNA2* and *IFNB1* expression, was also verified in tumors after PBMC transplantation (Fig. 7G and H). These results suggested that PTBP2 exerted antitumor effects by reeducating monocytes in mice in vivo.

### High *IFNA2* and *IFNB1* expression is associated with a favorable NB outcome

To correlate the above results with physiopathology in the clinic, we then measured the expression of *IFNA2* and *IFNB1* in a cohort of NB tumors with UP or FP (*n* = 10 in each group). Elevated *IFNA2* and *IFNB1* levels were detected in the FP-NB group compared to the UP-NB group (Fig. 7I). To sum up, these data further demonstrated that PTBP2 is a promising prognostic indicator and a potential therapeutic target.

## Discussion

Monocytes have been reported to be mainly recruited and activated by tumor-derived signals such as chemokines, cytokines, and other endogenous signals [6,24]. For high-risk NB children, preclinical and clinical evidence suggests that targeting the TME has been reported to benefit a subgroup of patients with NB [25,26]. The protumoral behavior of monocytes and TAMs has made them attractive therapeutic targets; this behavior includes inhibiting monocyte recruitment, decreasing the number of TAMs, and functional/phenotypic reprogramming [27]. NB cell has been reported to attract circulating monocytes and form an immunosuppressive TME [28]. Moreover, interleukin-1β (IL-1β) and tumor necrosis factor-α (TNF-α) released by NB cell-repolarized M1-Mϕs can also drive tumor cell proliferation through regulating arginine metabolism [8]. However, the key mediator of NB cells to monocytes polarization is still unclear. As reported previously, NB has ability to educate circulating monocytes into an anti-inflammatory phenotype, to exert immunosuppressive effect on lymphocyte cells [28]. Collagen-endocytosing TAMs derived from CCR2^+^ inflammatory monocytes recruited to and altered the TME through endocytic collagen turnover, which further centrally engaged in collagen degradation and promote invasive tumor growth [29]. In our study, we previously found that CD209^+^CD36^+^ monocytes were more abundant in mediastinal NB samples at advanced stages compared with earlier stages, while the CD80^+^CCR7^+^ subset abundantly existed in the early stage and demonstrated that classic monocytes were necessary for Mϕ differentiation and infiltration, to sustain the inflammatory state in an early mediastinal NB environment.

We further identified the alternative splicer PTBP2 from proteomic analysis of NBs with distinct prognoses and verified that monocytes and Mϕs polarized by PTBP2-treated NB cells also act back to regulate tumor cell proliferation. As mentioned previously, PTBP2 is expressed at much higher levels than PTBP1 is in the brain and controls an alternative splicing program during neuronal differentiation [18,19]. Considering the similar progenitors, we reasoned that the 2 splicing factors might perform a similar function on NB. Interestingly, GO and KEGG analyses of the proteomic data of NB samples revealed alternative mRNA splicing pathways. Moreover, PTBP2, rather PTBP1, in mediastinal NB with a favorable outcome was also found to be abundantly expressed on the basis of sequence data, hinting that PTBP2-mediated alternative splicing exerted a tumor-inhibiting effect in NB. Bioinformatic analysis of datasets from several publicly available children and nerve-related tumors also revealed that high PTBP2 expression predicted better outcomes, especially in NB. Therefore, functional and mechanistic studies are valuable to further clarify whether PTBP2 could be used as an independent predictor for the prognosis of NB.

A targeted functional study on PTBP2 in NB cell lines seems contradictory, in that PTBP2 in NB cell lines not only had minimal effects on cell viability but also showed a promigration tendency for NB cells. Considering that the interactions of the TME and tumor cells have been demonstrated to drive the malignant progression of various cancers, our analysis of immune cell infiltration in the NB-TME highlighting monocytes was positively correlated with NB prognosis. Moreover, CMs from coculturing monocytes with PTBP2-overexpressing NB cells significantly inhibited the proliferation and migration of NB cells in vitro and in vivo, which was consistent with bioinformatic analysis. Therefore, we then shifted our attention to monocytes and Mϕs in the TME affected indirectly by PTBP2-induced NB cells.

We first observed that PTBP2-stimulated CCL5 secretion was responsible for monocyte chemotaxis in the TME. CCL5 is known as a potent chemoattractant for monocytes, T helper cells, and eosinophils [30,31], and CCL5 can bind the chemokine receptors CCR1, CCR3, and CCR5 on the cell surface [32]. In addition, the CCL5/CCR5 axis is the main actor during tumor progression. Preclinical studies in various cancer models have demonstrated CCL5 as a double-edged sword in cancer. On the one hand, overexpression of CCL5 or CCR5 in cancer cells has been found to be associated with poor prognosis [33,34]. In addition, CCL5 also enhances antitumor immunity by recruiting antitumor T cells and dendritic cells to the TME and therefore promotes the immunotherapy response in different tumor types [35,36]. In our study, although PTBP2-stimulated CCL5 may be responsible for NB cell migration, it also has significant chemotactic activity for monocytes and Mϕs. Furthermore, CCL5-blocking antibodies have been shown to significantly suppress the migratory effect of PTBP2-induced NB cells on monocytes and Mϕs. However, CCL5 and anti-CCL5 antibodies rarely affect the tumor-inhibiting effect induced by PTBP2, leading us to hypothesize that PTBP2-attracted monocytes and Mϕs may exert their main antitumor effects through other mediators.

The interplay between human NB cells and monocytes/Mϕs was further clarified. RNA-seq data and mechanistic studies of monocytes verified that PTBP2-treated NB cells shaped monocytes/Mϕs to a classical phenotype by activating the IFN-I pathway, which had no obvious correlation with the CCL5/CCR5 axis. IFN-I signaling functions during the whole immune response by affecting innate antigen-presenting cells (APCs) to T cell responses [37]. Reducing IFN-I production in APCs becomes refractory to subsequent Toll-like receptor stimulation and IFN production [38]. Inflammatory IFN-I signals are critical and modulate the stimulatory capacity of Mϕs to prime a proinflammatory state and drive antitumor effects during the initial stages of cancer [39]. However, IFN-I responses can drive both tolerogenic and inflammatory immunity depending on the timing, cells present and IFNα/β subtypes [40,41]. Our study in NB cells supported that PTBP2-induced IFN-I signals are responsible for educating monocytes/Mϕs to an inflammatory phenotype, which also provides a validated explanation for PTBP2 in inhibiting NB growth via monocytes/Mϕs.

The driving mechanisms of how PTBP2 affects separate events, including CCL5-mediated chemotaxis and monocyte/Mϕ repolarization in the NB microenvironment, remain unclear. It is unadvisable for us to link the downstream genes of PTBP2 to IFN-I and immune-related signals from the transcriptomes of PTBP2-treated NB cells. However, when analyzing AS events specific to PTBP2 silencing, we found a series of inflammatory genes expressed in accordance with certain splicing patterns. Dysregulated RNA splicing can lead to tumorigenesis by altering tumor-related genes [42]. Therapeutics such as small molecules targeting components of the RNA splicing machinery have been developed and tested in clinical trials with varying outcomes [43,44]. By far, it is unknown whether and what changes in RNA splicing patterns are associated with NB cell viability in response to PTBP2 alteration. According to our results, we found an alternative splicing switch in IRF9 mRNA and confirmed that knocking down PTBP2 effectively accelerated the exon 6-7-8 region of IRF9 splicing into exons 6-8, which caused a deactivated alternative splicing switch and resulted in a loss of the full-length protein. IRF9 is a well-known component of the ISGF3 complex, along with phosphorylated STAT1 and STAT2, which move into the nucleus by activating and promoting transcription of many ISGs. Interestingly, we observed a dramatic increase in the protein levels of STAT1 and the ISGF3 complex, as well as IFNα/β gene induction upon PTBP2 and IRF9 overexpression, without IFN treatment. PTBP2-knockdown-induced exon 7 skipping not only regulated IRF9 transcription but also played roles in CCL5 and IFN-I signaling, as well as monocyte/Mϕ repolarization. This switch not only demonstrated a central role of PTBP2 in IRF9 alternative splicing and biogenesis but also highlighted that the full-length role of IRF9 in the downstream immunoregulatory transcriptional program was dependent on STAT1-induced ISGF3 formation during NB progression.

We hypothesized that CCL5 and IFN-I signals are simultaneously induced by PTBP2 and that they might share the unique upstream regulator of PTBP2. STAT1 was determined to be regulated by PTBP2 and downstream IRF9. Second, STAT1-induced ISGF was critical for PTBP2-mediated downstream immunoregulation in NB cells. Finally, it was predicted to have several binding activities in the region of CCL5 promoters. According to our studies, we verified that STAT1 accelerated the transcriptional activity and expression of CCL5 by binding to its promoter region mainly at −131 to −117 bp and −273 to −259 bp and revealed the inflammatory role of the STAT1-CCL5 axis in PTBP2 regulation.

The immune role of PTBP2 screened by proteomic profiling provided new insight into NB immunobiology. The superficially straightforward proinflammatory circuit has given way to an intricate, highly ordered, and concurrent network of immunity that is poorly understood, especially in NB. In summary, our study identified PTBP2 as an independent prognostic factor and predicted good outcomes in patients with NB or human PBMC-transplanted B-NDG (NOD. CB17-*Prkdcscid Il2rgtm1*/Bcgen) mice. Functionally, PTBP2 in NB cells has a certain effect on circulating monocytes and their progeny TAMs through activated chemotactic and IFN signaling pathways to inhibit NB growth and dissemination. Mechanistically, PTBP2 prevents IRF9 alternative splicing and upregulates STAT1/CCL5 and the STAT1/ISGF/IFN-I axis to induce monocyte chemotaxis and sustain monocytes/Mϕs in a proinflammatory phenotype (Fig. S10). These findings define a critical component of PTBP2-induced monocytes/Mϕs in NB progression. Our results suggest that targeting PTBP2 may represent a novel strategy for NB immunotherapy.

## Materials and Methods

### Tandem mass tag proteomic analysis and identification

We selected 5 pairs of NB patients with unfavorable (UP) or favorable prognosis (FP) for proteomic characterization. Whole proteins were extracted and hydrolyzed into peptides by trypsin, and all the peptides were labeled by Tandem Mass Tag (TMT) reagent (Thermo Fisher Scientific, Waltham, MA, USA), and graded by an Agilent 1260 Infinity II HPLC system. The chromatographic column was equilibrated and loaded by samples for separation, then eluted by an Easy nLC system, and analyzed by a Q Exactive Plus mass spectrometer (Thermo Fisher Scientific, Waltham, MA, USA). The original atlas files were then transformed into .mgf files, which were submitted to the MASCOT 2.6 server for database retrieval through the built-in tool of the software. Then, the database search files (.dat files) were sent back to the software through Proteome Discoverer 2.1 (Thermo Fisher Scientific, Waltham, MA, USA) and filtered according to a false discovery rate of <0.01 standard to obtain highly reliable qualitative results. Pathway analysis was conducted using the KEGG database. Fisher’s exact test was used to identify the enriched pathways by comparing the number of differentially expressed proteins and total proteins correlated to pathways.

### Cell culture and isolation

Human HUVECs and the NB cell lines SK-N-BE, SK-N-AS, Kelly, and SK-N-SH were obtained from the American Type Culture Collection (Manassas, VA, USA). SK-N-BE was maintained in a mixture of Eagle's minimal essential medium (EMEM) and F12 (1:1), SK-N-SH was maintained in EMEM. HUVECs, SK-N-AS, and Kelly cells were maintained in RPMI-1640. All types of media consisted of 10% fetal bovine serum (FBS) and 1% penicillin/streptomycin. Cells were all incubated at 37°C with 5% CO_2_ and tested without mycoplasma contamination. All types of cells were authenticated via short-tandem repeat profiling.

Human purified monocytes were isolated from PBMCs by anti-CD14-positive selection (#130-050-201, Miltenyi Biotec, Bergisch Gladbach, Germany) and cultured in RPMI 1640 medium consisting of 10% FBS with macrophage colony-stimulating factor (20 ng/ml; #30025, PeproTech, Rocky Hill, USA) for differentiation. Mature Mϕs were acquired after 7 to 10 days and ready for subsequent procedures.

Total T cells were obtained from PBMCs by a T Cell Negative Isolation Kit (#130-096-535, Miltenyi Biotec, Bergisch Gladbach, Germany) and cultured in T cell expansion medium (ImmunoCult-XF, STEMCELL Technologies, Vancouver, Canada) with CD2/CD3/CD28 beads and IL-2 (10 ng/ml) for stimulation and expansion.

### NB tissue samples

Frozen NB tissues were surgically resected from Guangzhou Women and Children’s Medical Center and classified by stage or prognosis, as indicated on the basis of the clinicopathological information. All the patients provided written informed consent before the collection of samples. Ethical approval was obtained from the institutional ethics committee. This study was conducted strictly according to the ethical standard of the World Medical Association Declaration of Helsinki.

### RNA-seq and bioinformatics analysis

Total RNA was extracted and treated with ribonuclease-free deoxyribonuclease I (#M6101, Promega, WI, USA). The RNA integrity number was calculated using the RNA Nano 6000 Assay Kit of a Bioanalyzer 2100 system (Agilent Technologies, CA, USA). All RNA samples had an RNA integrity number of >8. The library fragments were then purified with the AMPure XP system (Beckman Coulter, Beverly, USA) and amplified by PCR. The cDNA library quality was quantified by using Qubit 2.0, detected on a Bioanalyzer 2100 (Agilent, USA), and then sequenced using an Illumina NovaSeq 6000 platform. Differentially expressed genes from the transcriptome data were determined by the DESeq2 R package (1.20.0), with a fold change of >2. GO and KEGG pathway enrichment analysis of the differentially expressed genes was implemented by the clusterProfiler R package (3.8.1). The AS event was analyzed by the variable of protein using rMATS (4.0.2) software.

For analysis of public datasets, RNA-seq-based gene expression data in children’s solid tumors, as well as glioblastoma, were obtained from the Gene Expression Profiling Interactive Analysis database for cancer genomics. For gene expression, *P* < 0.05 was used as the cutoff.

### IHC assays

NB tissue samples and mouse tumor samples were fixed and embedded for IHC staining as previously described [45]. After blockade, the sections were separately incubated with mouse anti-PTBP2 antibody (#sc-376316, Santa Cruz Biotechnology, CA, USA), rabbit anti-PTBP1 antibody (#ab133734, Abcam, Cambridge, MA, USA), rabbit anti-human CCL5 antibody (#12000-1-AP, Proteintech, Chicago, IL, USA), rabbit anti-Ki67 antibody (catalog no.12202, Cell Signaling Technology, Beverly, USA), mouse anti-human CD68 antibody (#ab201340, Abcam), and anti-Twist antibody (#25465-1-AP, Proteintech) at 4°C overnight, hybridized with biotinylated anti-rabbit/mouse immunoglobulin at room temperature for 1 h, and visualized by diaminobenzidine (GK500705, Dako, Glostrup, Denmark). Three to 5 representative fields of each section were captured and analyzed. The average positive ratio was defined by the symbol and color and analyzed using Image-Pro Plus 6.0 software (Media Cybernetics, Rockville, MD, USA).

### IF assay

Briefly, the fixed cells were permeabilized and incubated with anti-PTBP2 antibody, anti-human STAT1 antibody (#10144-2-AP, Proteintech, Chicago, IL, USA), and anti-IRF9 antibody (#14167-1-AP, Proteintech, Chicago, IL, USA) separately. They were further incubated with anti-mouse or rabbit Alexa Fluor secondary antibodies (#4414 or #4409, Cell Signaling Technology, Beverly, USA). The nuclei were subsequently visualized with 4′,6-diamidino-2-phenylindole (DAPI) staining (#P0131, Beyotime, Nanjing, China). Images of the cells were captured by a confocal microscope (Leica SP8, Germany) equipped with a 20× objective and then analyzed using Image-Pro Plus 6.0 software.

### Cell transfection

Human cDNAs of the CCL5 promoter region, including full length, Pro 3 (142-bp upstream), Pro 3 + Pro 2 (282-bp upstream), and Pro 1 + Pro 2 + Pro 3 (961-bp upstream) cDNAs of the CCL5 promoter region, were cloned and ligated into a pGL4.10 vector. The full-length cDNAs of human PTBP2, STAT1, and STAT2 were cloned and ligated into a pcDNA3.1 vector. The short hairpin–mediated RNA (shRNA) sequences targeting human PTBP2 were inserted into the vector pDKD-CMV-Puro-U6-shRNA. Cells were transfected by using Lipofectamine 3000 (Invitrogen, Carlsbad, CA, USA). The scrambled shRNA sequence was a control for PTBP2 knockingdown, while the empty vector was a control for PTBP2 overexpression. All plasmids were provided by OBiO Technology Inc. (Shanghai, China). The knockdown or overexpression efficiency was further validated by the mRNA and protein levels of targeted genes. siRNAs targeting IRF9, STAT1, and STAT2 were used separately to transiently knockingdown the indicated genes in NB cells (RiboBio Co., Ltd, Guangzhou, China), and a control transfected with scrambled sequence was used in the study. The sequences of all the knocked down targeted genes are shown in Table S1.

### Flow cytometry analysis

To detect the phenotype of monocytes or Mϕs, isolated cells were treated with the indicated CM for 48 h and subjected to flow cytometry analysis by staining with phycoerythrin (PE)/Cyanine 7 anti-human CD45 (#368532), APC anti-human CD209 (#330107), fluorescein isothiocyanate anti-human CD80 (#305206), PerCP anti-human CD14 (#325032), PE anti-human CD11b (#301305), and PE/Cyanine7 anti-human CCR5 (#359108) from BioLegend (San Diego, CA, USA). The staining procedures followed those of the manufacturer’s instructions.

### Cell-based CM

We generated 2 types of CMs during our study: For the monocyte and Mϕ chemotaxis assay, NB-CMs were from NB cell lines, which subjected to PTBP2 overexpression or knockingdown for 48 h and then placed each group of CM, as well as fresh medium (1:1) in the lower chambers of Transwell system; for functional study of NB cells, co-CMs were generated from cocultured system (upper: monocytes; lower: NB cells with PTBP2 alteration for 48 h).

### Cell proliferation viability

Cell proliferation viability and colony formation assays were performed according to the manufacturer’s instructions. Briefly, NB cells were plated in 96-well plates or 6-well plates overnight and treated as indicated. The cell viability index was obtained by calculating the CCK-8-induced (Dojindo, Japan) absorbance of cells in 96-well plates at 450 nm or clone counts in 6-well plates of each group.

### Cell cycle and apoptosis analysis

Cell cycle analysis was performed with cell cycle detection kit (#KGA511, KeyGen, Nanjing, China) according to the manufacturer’s instructions. Briefly, co-CM-treated NB cells were fixed with 75% ethanol and then stained with propidium iodide; the cell cycle transition of each group was analyzed by flow cytometry and calculated by ModFit software (3.1).

For cell apoptosis analysis, co-CM-treated NB cells were harvested and washed with phosphate-buffered saline and then resuspended in staining buffer plus propidium iodide and annexin V- Alex Fluor 647 (AP006-100, ES Science, Shanghai, China). The percentages of cells undergoing apoptosis were evaluated by flow cytometry.

### Cell migration and chemotaxis assays

For the cell migration assay, cells (5 × 10^4^ per well) with 1% FBS were seeded in the upper inserts (Corning, NY, USA) and then exposed to media consisting of 10% FBS or the indicated CM in the lower chambers (inserts with 8-μm pore size). Cells migrating to the lower surface of the inserts were counted after 12 h and analyzed using Image-Pro Plus 6.0 software.

For the monocyte chemotaxis assay, purified monocytes (1 × 10^6^ per well) were seeded in the upper chambers of a 24-well Transwell plate (Corning, NY, USA) and exposed to media containing the indicated CMs in the lower chambers (inserts with 0.4-μm pore size) for 12 h. The chemotaxis of monocytes was analyzed by counting the number of migrated cells.

### Immunoblotting

Immunoblotting was performed according to standard procedures. Total proteins were extracted from treated cells lysed with ice-cold radioimmunoprecipitation assay buffer containing protease and phosphatase inhibitors. The amounts of proteins were quantified using a bicinchoninic acid (BCA) protein assay kit (Thermo Fisher Scientific, Waltham, MA, USA). The primary antibodies used included anti-PTBP2, anti-PTBP1 antibody, anti-IRF9, anti-STAT1, anti-vinculin (#13901, Cell Signaling Technology, Beverly, USA), anti-STAT2 (#16674-1-AP, Proteintech, Chicago, IL, USA), anti-β-actin (#4790, Cell Signaling Technology, Beverly, USA), and anti-histone H3 (#4499, Cell Signaling Technology, Beverly, USA). All the bands were visualized by enhanced chemiluminescence, replicated at least 3 times, and quantified by ImageJ analysis software.

### RT-PCR analysis

Total RNA was obtained using an RNA extraction kit (#RN001, ES Science, Shanghai, China) and reverse-transcribed to cDNA according to the manufacturer’s instructions (#RR036A-1, TAKARA, Japan). The relative expression of the identified genes was determined and measured by quantitative RT-PCR assays (ABI Q6 System, Applied Biosystems). The expression levels of all the targeted genes were normalized to those of β-actin, and the fold changes were calculated via the 2^−ΔΔCt^ comparative method. The sequences of the specific primers used are shown in Table S2.

### Enzyme-linked immunosorbent assays

The supernatants from treated NB cells or plasma from the blood were collected and diluted for CCL5 measurement using a human CCL5 SimpleStep ELISA (enzyme-linked immunosorbent assay) Kit (#ab174446, Abcam, Cambridge, MA, USA). Briefly, 50 μl of samples were added to appropriate wells, followed by the addition of 50 μl of antibody cocktail to each well, after incubation at room temperature for 1 h. Each well was washed 3 times with 350 μl of wash buffer. Stop solution was added and incubated for 10 min at room temperature before the addition of 100 μl of stop solution prior to measurement of the absorbance at 450 nm.

### Cytokine measurements

Cytokines secreted by PTBP2-knockingdown-induced NB cells and control cells were measured and quantified by inflammatory cytokine array measurement (AAH-CYT-G5-4, RayBiotech) according to the manufacturer’s instructions. The measured cytokine panels are listed in Fig. S1.

### CLIP and ChIP assays

NB cells with PTBP2 alterations were irradiated with 254-nm ultraviolet light (400 mJ/cm^2^). The cells were pelleted and lysed by NP-40 lysis buffer supplemented with protease inhibitors, 1 mM dithiothreitol, and ribonuclease (400 U/ml) inhibitor for 60 min at 37°C. Afterward, 30 μl of deoxyribonuclease I was added at 37°C for 10 min, and the reaction was stopped by 4 μl of 0.5 M EDTA. Total RNA was fragmented by micrococcal nuclease (#10011, Cell Signaling Technology, Beverly, USA) digestion, while the input RNA was separated by electrophoresis on a 1% agarose gel to ensure that the majority of RNA was digested to 1 to 5 nucleosomes (150 to 900 bp). Then, 40 μl of pre-equilibrated beads (HY-K0202, MedChemExpress, New Jersey, USA) was incubated with 10 μg of normal immunoglobulin G or PTBP2 antibody overnight at 4°C. The beads were washed, and 1 ml of cell lysates was added. The mixtures were incubated for 2 h at 4°C and dephosphorylated by FAST AP (#EF0654, Thermo Fisher Scientific, Waltham, MA, USA) for 1 h at 37°C. The protein–RNA complexes were further eluted from the beads and collected for RNA extraction, after which quantitative PCR analysis was performed.

For the ChIP assay, a Simple ChIP kit (#66816, Cell Signaling Technology) was used to detect DNA immunoprecipitated with STAT1 according to the manufacturer’s protocol. The complexes were further reacted with Simple CHIP qPCR Master Mix (#88989, Cell Signaling Technology) for quantitative real-time PCR assays.

### Co-IP assays

Anti-IRF9, anti-STAT1, and anti-STAT2 were used for co-IP assays as described previously [45]. The immune precipitants were detected by immunoblotting. Anti-rabbit and mouse immunoglobulin G (#3900 and #3420, Cell Signaling Technology, Beverly, USA) were used as negative controls.

### Luciferase reporter assays

NB cells were plated in 96-well plates, transfected with plasmids containing the CCL5 promoter and inducible luciferase and subjected to the treatments as indicated. The luciferase activity was evaluated by the Steady-Glo Reporter Assay (#E2510, Promega, WI, USA) as per the manufacturer’s instructions.

### In vivo study

Metastatic NB models were established by tail vein injection with SK-N-SH cells (5 × 10^5^/mice) expressing inducible luciferase reporter in immunodeficient B-NDG (NOD. CB17-*Prkdcscid Il2rgtm1*/Bcgen) mice. All the NB mice were randomly assigned into 1 of 6 groups (*n* = 12 per group): Ctrl, Sh PTBP2, PTBP2, Ctrl + PBMC, shPTBP2 + PBMC, and PTBP2 + PBMC. After 2 weeks, mice with PBMC group were transplanted 5 × 10^6^ human PBMCs by tail vein injection. Tumor size was monitored by luciferase imaging using In Vivo Bruker FX PRO. Luciferase intensity was captured and measured, and metastasis nodes were also counted and analyzed. All the mice were sacrificed by transplanting PBMCs for 3 weeks, and tumor tissues were collected for further pathological analysis. All animal experiments meet the standard and approved by the Institutional Animal Care and Use Committee of Sun Yat-Sen University Cancer Center.

### Statistical analysis

All Western blots, IF, and IHC images are representative results of at least 2 independent biological replicates. Correlations between PTBP2 and CD163 expression were determined with Pearson’s correlation analysis. All the bar graphs show the means ± SDs. Statistical calculations were conducted from at least 3 independent experiments and analyzed by Student’s *t* test (unpaired, 2-tailed) or one-way analysis of variance (ANOVA) using GraphPad Prism 8.0.1 software (GraphPad, La Jolla, CA, USA). A *P* < 0.05 indicates statistical significance. In the figures, the following are used: *P < 0.05, **P < 0.01, and ***P < 0.001.

## Data Availability

All the data supporting the findings of this work are available within the article and its Supplementary Materials.
