# Supplementary material for: PTBP2-Mediated Alternative Splicing of IRF9 Controls Tumor-Associated Monocyte/Macrophage Chemotaxis and Repolarization in Neuroblastoma Progression
Source: Research (Wash D C). 2023 Jan 30;6:0033. doi: 10.34133/research.0033 (PMC10076020; doi:10.34133/research.0033)
Supplement: Supplementary 1 — Figs. S1 to S10. Tables S1 to S3. [file research.0033.f1.docx]

**Supplementary Materials for**

PTBP2-mediated alternative splicing of IRF9 controls tumor-associated monocyte/macrophage chemotaxis and repolarization in neuroblastoma progression

Jue Tang^1*^, Jing He^1*^, Huiqin Guo^1,2*^, Huiran Lin^3^, Meng Li^1^, Tianyou Yang^1^, Hai-Yun Wang^1^, Di Li^1^, Jiabin Liu^1^, Le Li^1^, Huimin Xia^1†^, Zhenjian Zhuo^1, 4†^ and Lei Miao^1†^

Correspondence to: miaoleimolly@126.com; [zhenjianzhuo@163.com](mailto:zhenjianzhuo@163.com); [xia-huimin@foxmail.com](mailto:xia-huimin@foxmail.com)

**This PDF file includes:**

**Figures. S1 to S10**

**Tables S1 to S3**

**
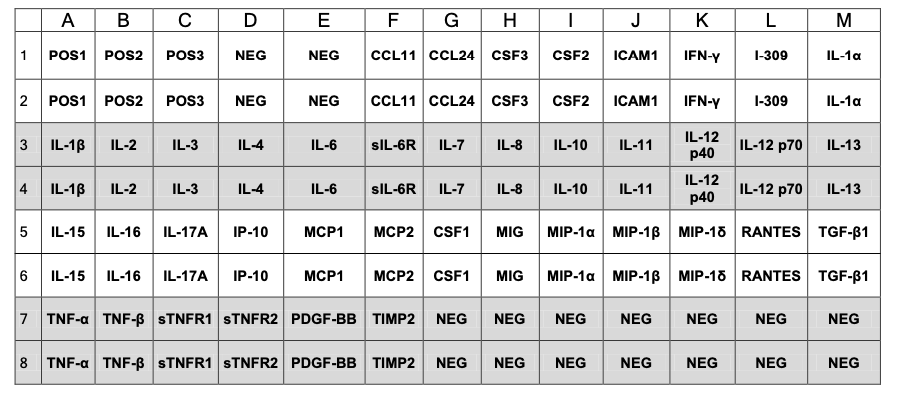
**

**Figure S1.** Panel of inflammatory cytokine arrays (AAH-CYT-G5-4).

**
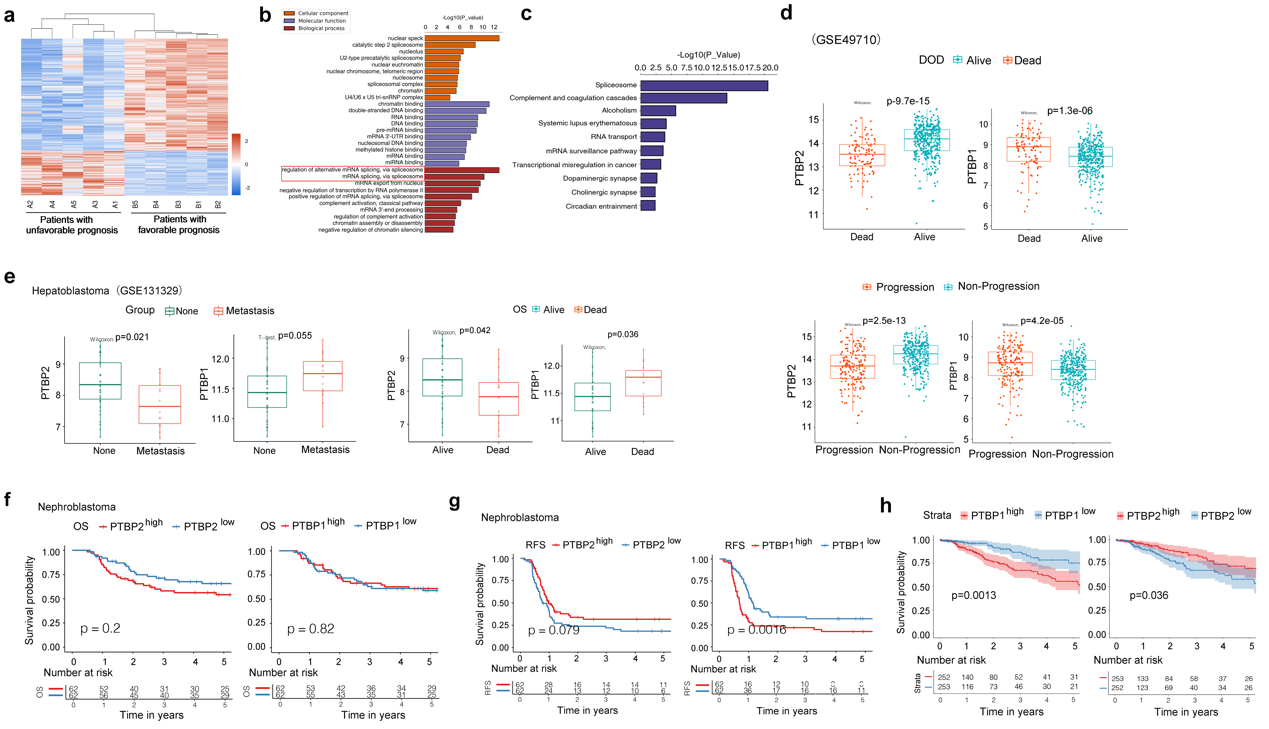
**

**Figure S2.** Expression differences of PTBP1 and PTBP2 in other children and in nerve-related tumors. **(a)** Heatmap of differentially expressed proteins from the quantitative proteomic profile in 10 NB tumors with UP or FP after chemotherapy. **(b, c)** GO and KEGG pathway enrichment analysis of the quantitative proteomic profile. **(d)** Bioinformatics analysis of PTBP1 and PTBP2 expression in the DOD and progression cluster from a publicly accessible NB database (GSE49710). **(e, f)** Bioinformatic analysis of PTBP1 and PTBP2 expression in hepatoblastoma with or without metastasis and OS levels from a publicly accessible database (GSE131329). **(g, h)** The OS and RFS of PTBP1 and PTBP2 in TCGA nephroblastoma database. **(i)** The RFS of PTBP1 and PTBP2 in TCGA database of low-grade glioblastoma.


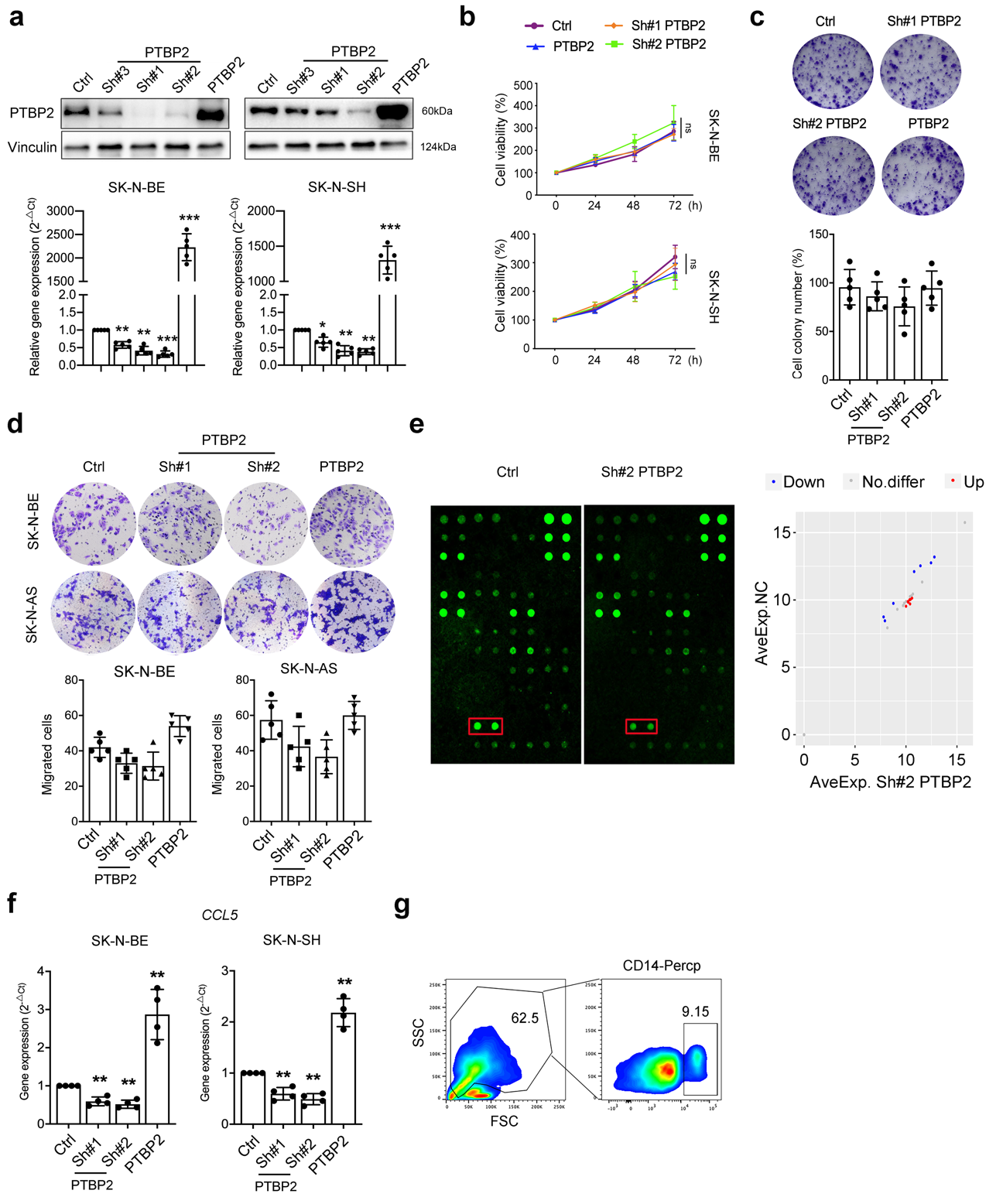


**Figure S3.** Function of PTBP2 in NB cells. **(a)** Immunoblot and RT–PCR analysis showing PTBP2 expression in SK-N-BE and SK-N-SH cells with PTBP2 knockdown or overexpression. The data are presented as the mean ± SD (n = 3). ^*^*p* < 0.05; ^**^*p* < 0.01; ^***^*p* < 0.001. **(b)** The proliferation rate of human NB cells with PTBP2 knockdown or overexpression was assessed by CCK-8 assay. **(c)** Colony formation assay of human NB cells subjected to PTBP2 alteration. **(d)** Transwell assay of human NB cells treated as indicated. **(e)** Left panel: Representative pictures of the cytokine array with or without PTBP2 treatment. Right panel: Scatterplot of cytokines with or without PTBP2 treatment. **(f)** mRNA expression of *CCL5* in SK-N-BE and SK-N-SH cells with PTBP2 alteration. The data are presented as the mean ± SD (n = 3). ^**^*p* < 0.01. Ctrl in a-d, f stands for the average of undistinguishable controls of scrambled sequence (for Sh-PTBP2) and empty vector (for PTBP2 overexpression). **(g)** CD14^+^ monocytes from human PBMCs were labeled and selected by flow cytometric analysis.


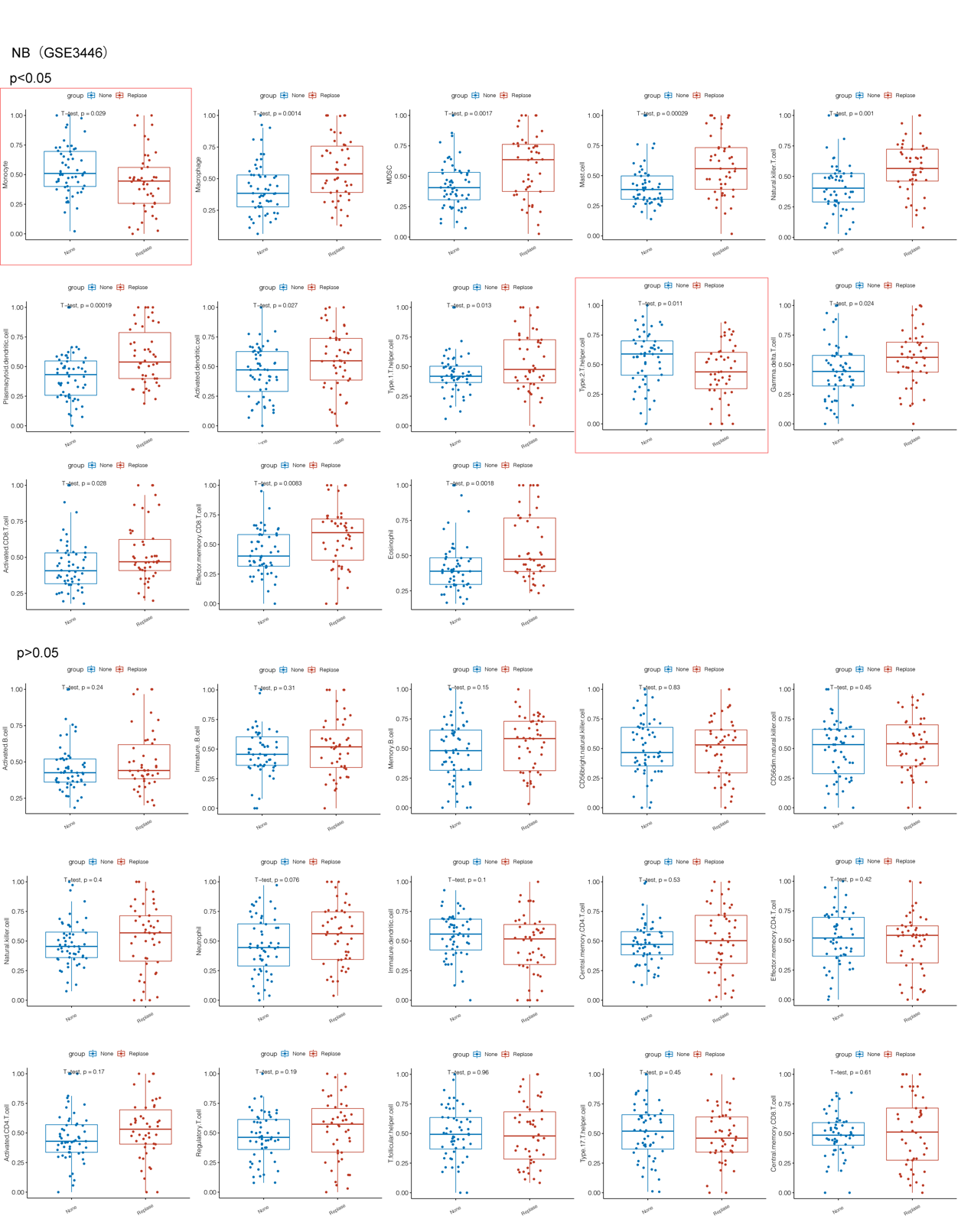


**Figure S4.** Bioinformatic analysis of immune cell infiltration in NB-TME (GSE3446).


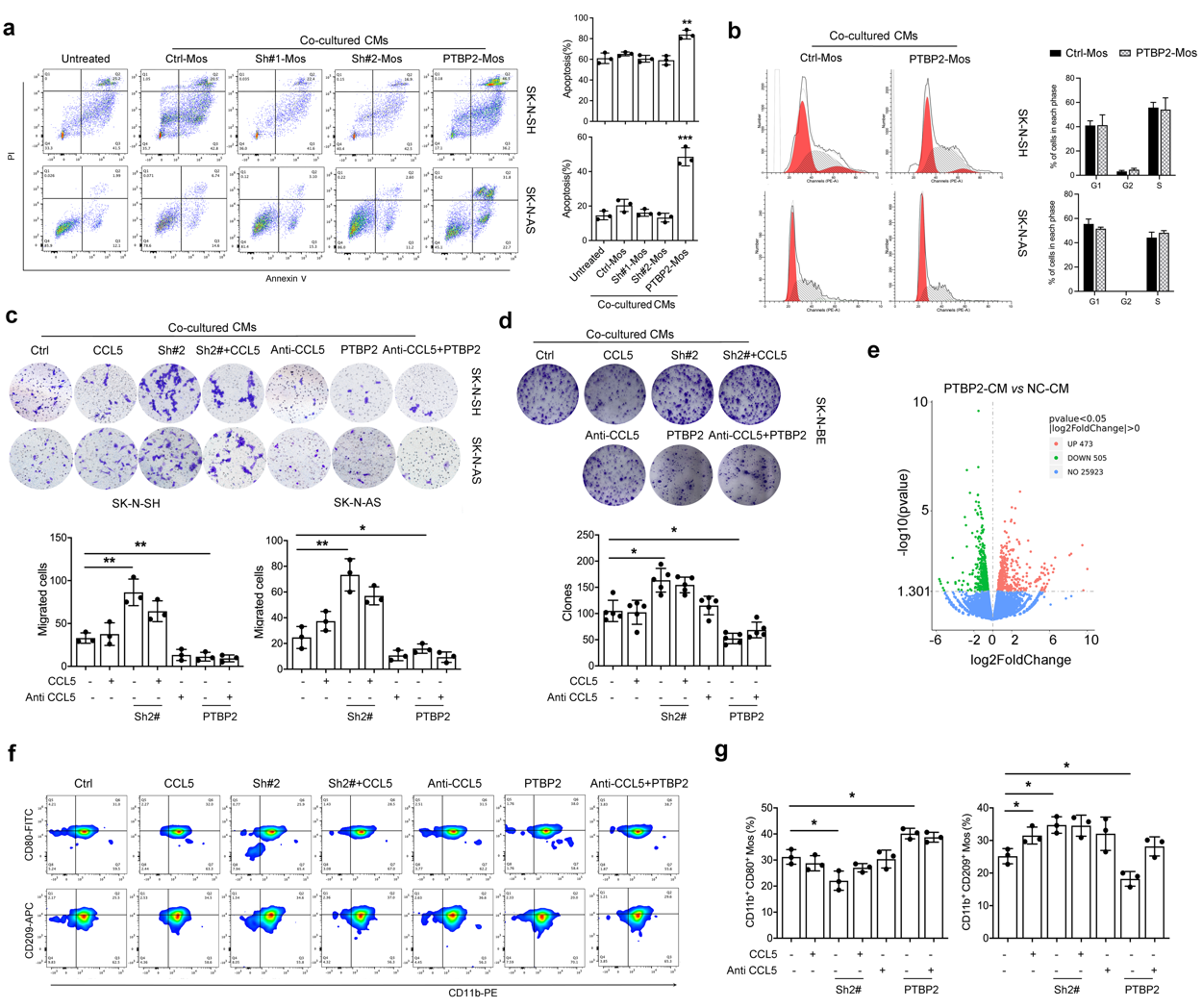


**Figure S5.** NB cell progression inhibited by PTBP2-treated monocytes is independent of CCL5. **(a)** Apoptosis assay and statistical analysis of SK-N-SH cells and SK-N-AS subjected to co-CM treatment as indicated. **(b)** Cell cycle assay and statistical analysis of SK-N-SH cells and SK-N-AS subjected to co-CM treatment as indicated. **(c)** Transwell assay and statistical analysis of SK-N-SH cells and SK-N-AS subjected to co-CM treatment as indicated. **(d)** Colony formation assay and statistical analysis of SK-N-BE cells subjected to co-CM treatment, as indicated. **(e)** Volcano plot of RNA-seq expression in PTBP2 overexpression-stimulated M𝜙s. **(f, g)** Representative flow cytometry plots of CD11b^+^CD80^+^ and CD11b^+^CD209^+^ monocytes treated as indicated. The quantification of the percentage is shown and analyzed in **d**. The data in a-d, g are representative of three independent experiments and presented as the mean ± SD; ^*^*p* < 0.05; ^**^*p* < 0.01. Ctrl represents the average of undistinguishable controls of scrambled sequences (for Sh-PTBP2) and the empty vector (for PTBP2 overexpression).


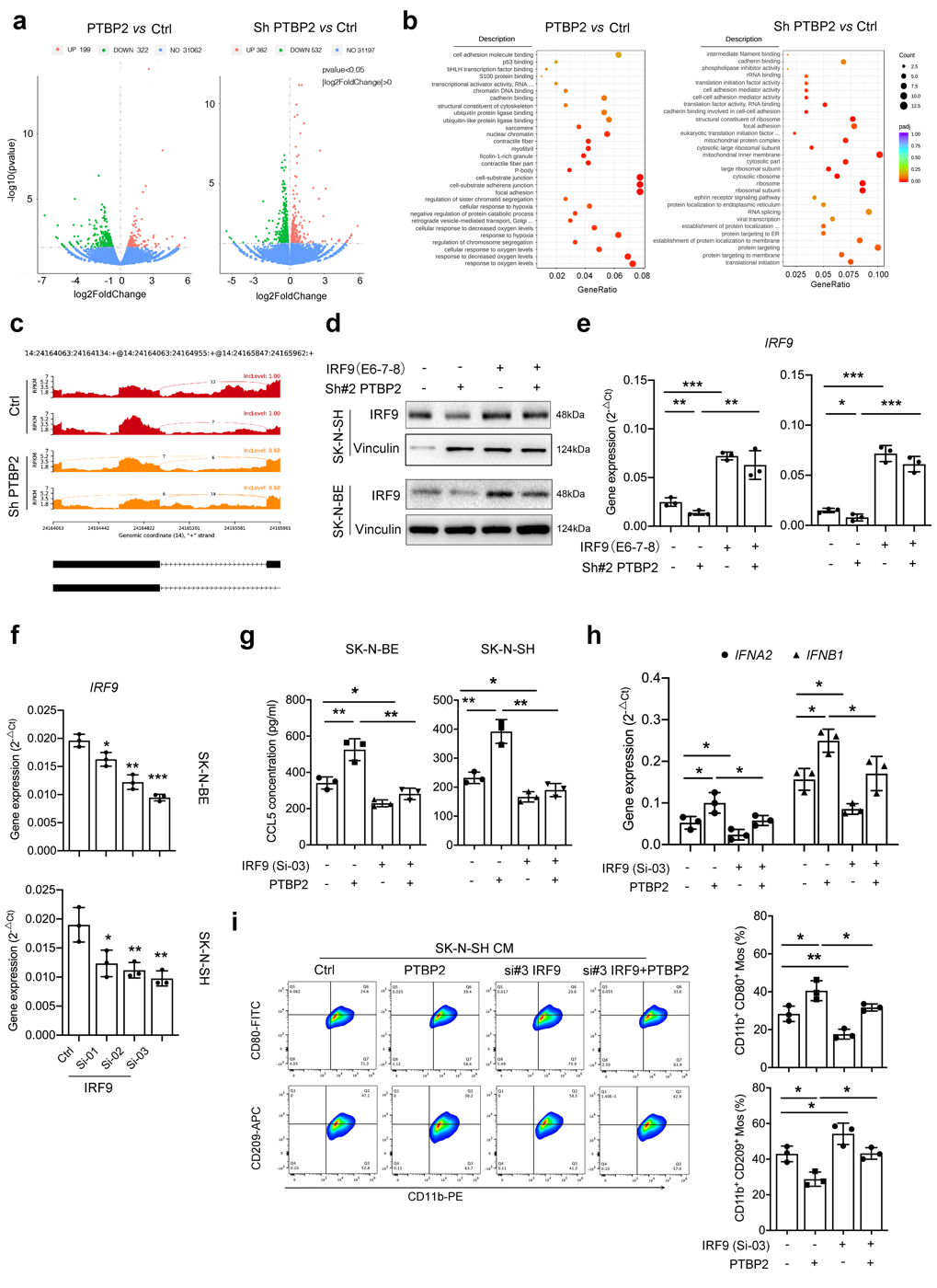


**Figure S6.** PTBP2-induced alternative splicing of IRF9 affects CCL5 and IFNα/β expression. **(a)** Volcano plot of RNA-seq expression in PTBP2-treated SK-N-SH cells. **(b)** KEGG pathway enrichment analysis of RNA-seq expression profiling, which was performed according to the GSEA method based on an empirical permutation test procedure. **(c)** Alternative splicing event of *IRF9* in the alternative 5’ splice site was determined from RNA-seq reads in the PTBP2 knockdown and control groups. **(d)** Immunoblot analysis showing IRF9 expression after treatment as indicated. Vinculin was used as a loading control. **(e)** RT–PCR analysis of *IRF9* treated as indicated. **(f)** RT–PCR analysis of *IRF9* with siRNAs treatment. **(g)** ELISA for CCL5 concentration in the supernatant of SK-N-BE and SK-N-SH cells treated as indicated. **(h)** RT–PCR analysis of *IFNA2* and *IFNB1* expression after treatment, as indicated. **(i)** Left panel: Representative flow cytometry plots of CD11b^+^CD80^+^ and CD11b^+^CD209^+^ monocytes treated as indicated. Right panel: Quantification of percentage in each group. The data in **e-i** are representative of three independent experiments and presented as the mean ± SD. ^*^*p* < 0.05; ^**^*p* < 0.01; ^***^*p* < 0.001. Ctrl represents the average of undistinguishable controls of scrambled sequences (for Sh-PTBP2) and the empty vector (for PTBP2 overexpression).


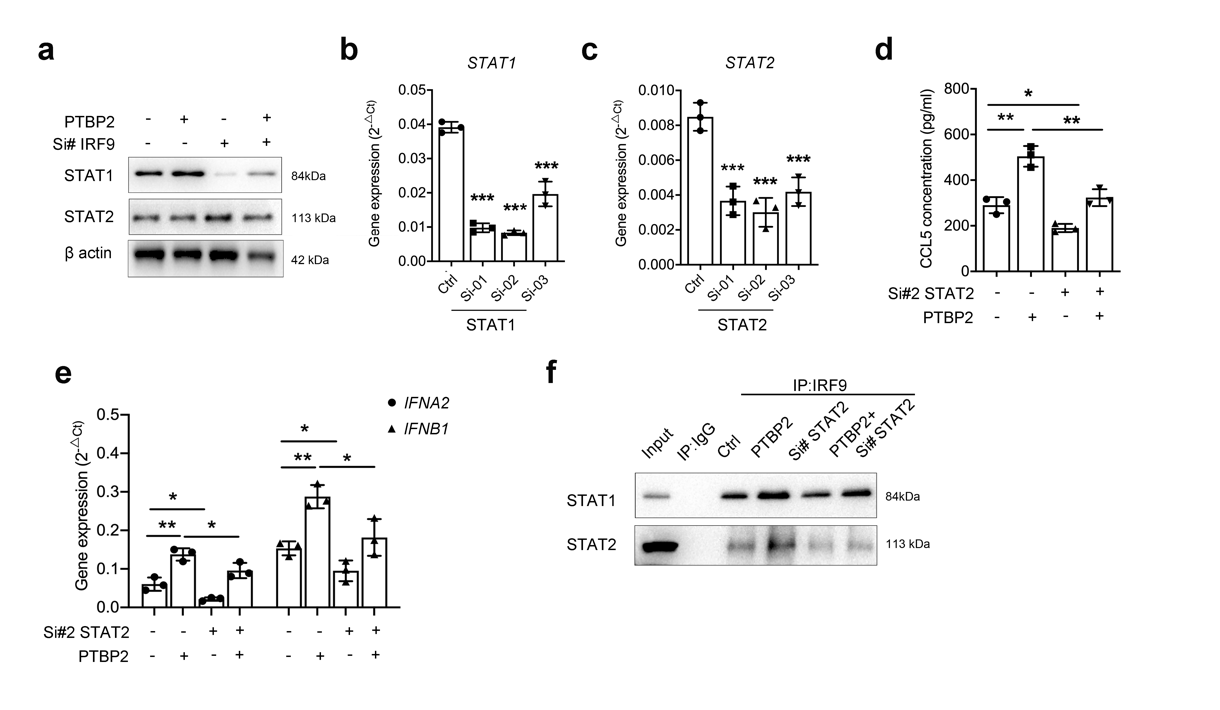


**Figure S7.** ISGF3 activation is responsible for PTBP2-induced IFNα and IFNβ expression. **(a)** Immunoblot analysis of STAT1 and STAT2 in SK-N-SH cells treated as indicated. β-Actin was used as a loading control. **(b, c)** RT–PCR analysis of *STAT1* and *STAT2* with siRNAs. **(e**) ELISA for CCL5 concentration treated as indicated. **(e**) RT–PCR analysis of *IFNA2* and *IFNB1* expression after treatment, as indicated. **(f)** Co-IP assays for SK-N-SH cells treated as indicated were performed to verify the interaction among IRF9, STAT1, and STAT2. The data in **a-f** are representative of three independent experiments and presented as the mean ± SD; n = 3 biologically independent samples. ^*^*p* < 0.05; ^**^*p* < 0.01; ^***^*p* < 0.001. Ctrl represents the average of undistinguishable controls for siRNAs.


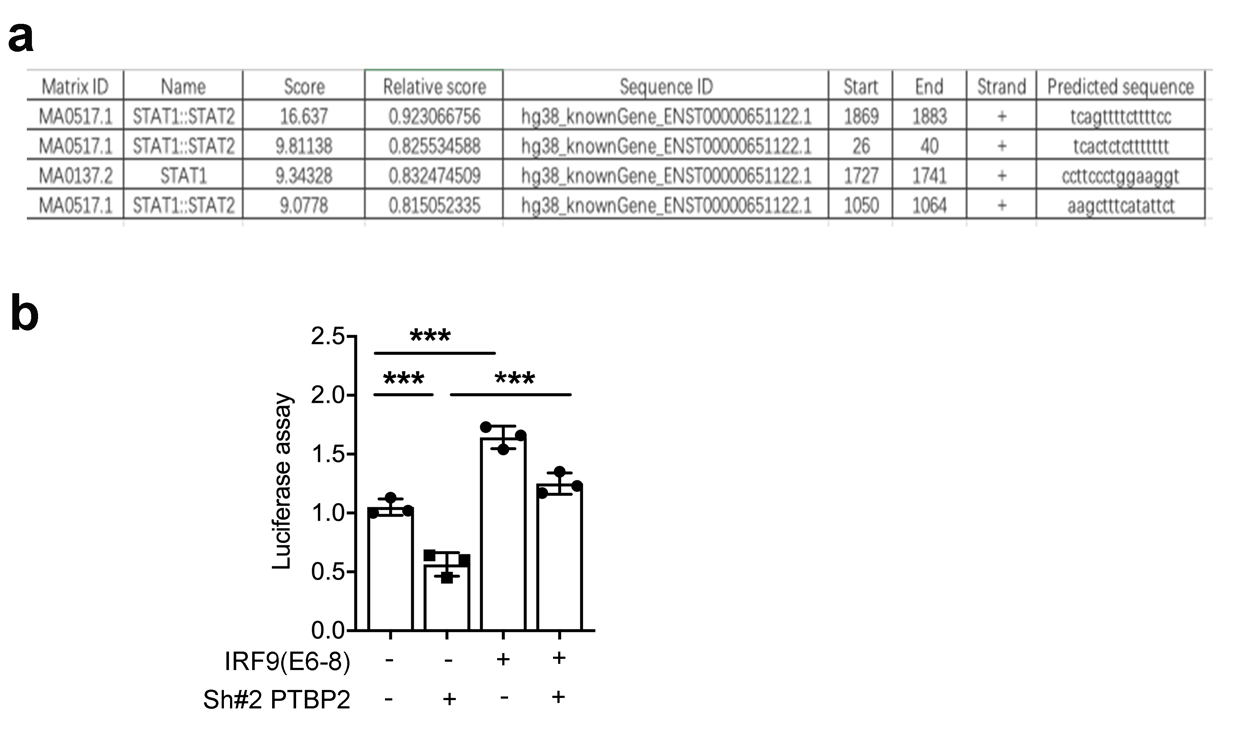


**Figure S8.** STAT1 binds to the promoter region of CCL5 and regulates its transcriptional activity. **(a)** The possible promoter binding regions of CCL5 by STAT1 were predicted by the JASPAR database (version 2020). **(b)** Transcriptional activity of CCL5 in SK-N-SH cells treated as indicated evaluated by luciferase assays. The data in **b** are representative of three independent experiments and presented as the mean ± SD; n = 3 biologically independent samples. ^*^*p* < 0.05; ^**^*p* < 0.01; ^***^*p* < 0.001. Ctrl represents the average of undistinguishable controls of scrambled sequences (for Sh-PTBP2) and the empty vector (for IRF9 overexpression).


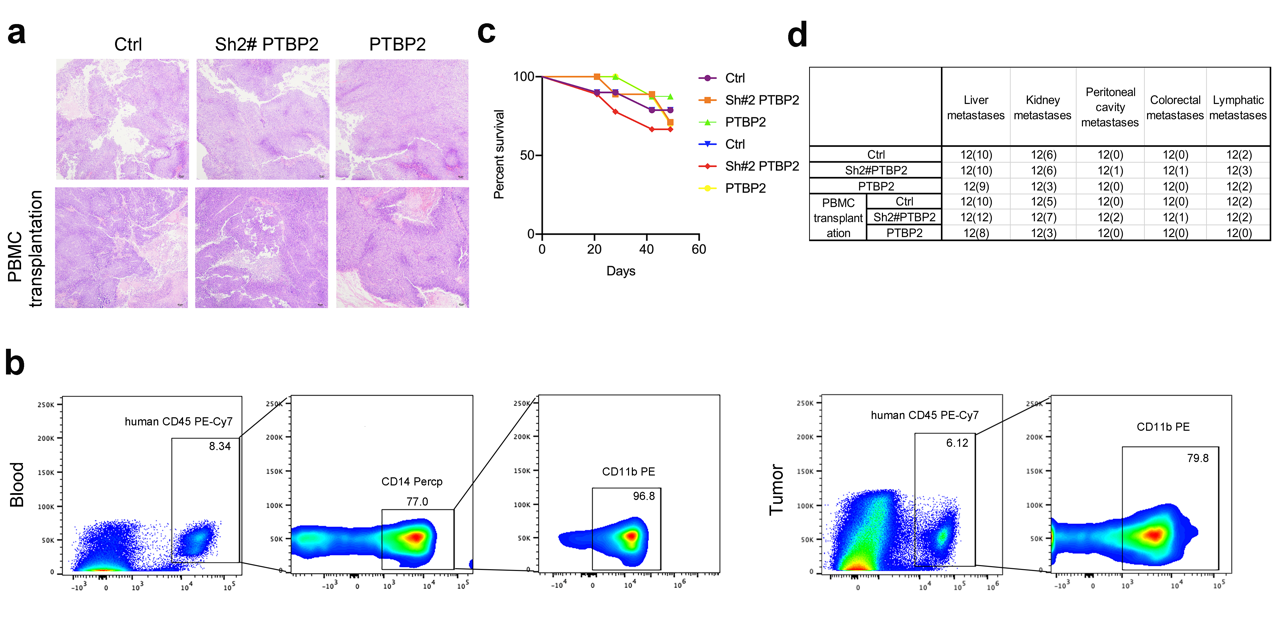


**Figure S9.** PTBP2 overexpression has antitumor effects on NB tumors through re-educating monocytes in mice. **(a)** Representative pictures of H&E staining in the NB model treated as indicated. The scale bars represent 50 µm. **(b)** Representative flow cytometric histogram of human monocytes and M𝜙s (CD45^+^CD14^+^CD11b^+^) isolated from mice. Ctrl represents a representative sample of undistinguishable controls of scrambled sequences (for Sh2-PTBP2) and the empty vector (for PTBP2 overexpression). **(c)** The survival rate of the NB model in each group was recorded. **(d)** The numbers of liver, kidney, peritoneal cavity, lymphatic nodes, and colorectal metastases treated as indicated were recorded.


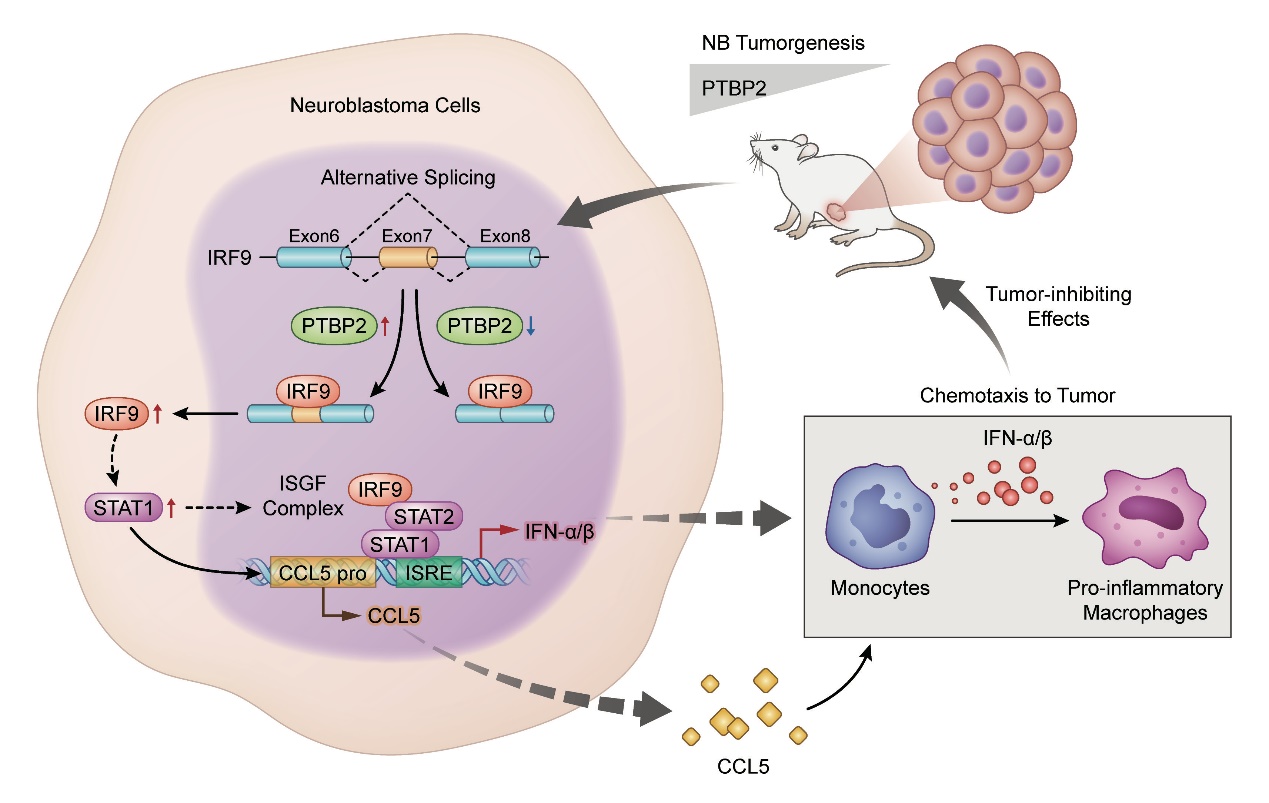


**Figure S10.** Possible molecular mechanisms of PTBP2 in neuroblastoma. PTBP2 prevents IRF9 alternative splicing, upregulates STAT1 to stimulate CCL5 and ISGF-dependent IFN-I secretion, to induce monocyte/M𝜙s chemotaxis and sustain monocytes in a proinflammatory phenotype.

| **Supplementary Table 1.** Sequences of ShRNAs and SiRNAs | | |
| --- | --- | --- |
| **Name** |  | **Sequences** |
| *Homo* *PTBP2* | Sh#1 | GCAGCTATTACTATGGTTA |
|  | Sh#2 | CCCTGTAACACTTGATGTT |
|  | Sh#3 | GCATTTGCCAAGGAGACAT |
| SC | Srcamble | TTCTCCGAACGTGTCACGT |
| *Homo STAT1* | Si#1 | CTGGATATATCAAGACTGA |
|  | Si#2 | GCACGCTGCCAATGATGTT |
|  | Si#3 | CATGCGGTTGAACCCTACA |
| *Homo STAT2* | Si#1 | GCAGCACAATTTGCGGAAA |
|  | Si#2 | GGACTGAGGATCCATTATT |
|  | Si#3 | GTGATAGAGCCCACACTAT |
| *Homo IRF9* | Si#1 | CCACCGAAGTTCCAGGTAA |
|  | Si#2 | GGAGCAGTCCATTCAGACA |
|  | Si#3 | CTCAGAAAGTACCATCAAA |

| **Supplementary Table 2.** Primers for RT-PCT analysis | | |
| --- | --- | --- |
| Name | Forward primer | Reverse primer |
| *Homo* β actin | CTACCTCATGAAGATCCTCACCGA | TTCTCCTTAATGTCACGCACGATT |
| *Homo* IL1B | AGCTACGAATCTCCGACCAC | CGTTATCCCATGTGTCGAAGAA |
| *Homo* CCL5 | AGCAGTCGTCCACAGGTCAA | CTGGGTTGGCACACACTTGG |
| *Homo* IFNB1 | GGCGACACTGTTCGTGTTGT | AGCCTCCCATTCAATTGCCAC |
| *Homo* IFNA2 | CTTGTGCCTGGGAGGTTGTC | GGTGAGCTGGCATACGAATC |
| *Homo* IRF9 | GCCCTACAAGGTGTATCAGTTG | TGCTGTCGCTTTGATGGTACT |
| *Homo* STAT1 | ATCAGGCTCAGTCGGGGAATA | TGGTCTCGTGTTCTCTGTTCT |
| *Homo* STAT2 | CCAGCTTTACTCGCACAGC | AGCCTTGGAATCATCACTCCC |
| *Homo* TNF | CCTCTCTCTAATCAGCCCTCTG | GAGGACCTGGGAGTAGATGAG |
| *Homo* PTBP2 | GCAACCGAGGAAGCAGCTATT | GCCTGAGCACGTTGGTTTAATG |
| *Homo* PTBP1 | AGCGCGTGAAGATCCTGTTC | CAGGGGTGAGTTGCCGTAG |
| *Homo* ELF2B4 | CTCTCACCTACCCCAGTACAG | ACGAAGCAGGGCAATACACC |
| *Homo* NONO | GGCAGGCGAAGTCTTCATTCA | TGGCAATCTCCGCTAGGGT |
| *Homo* SFPQ | AGCGATGTCGGTTGTTTGTTG | AGCGAACTCGAAGCTGTCTAC |
| *Homo* MATR3 | ATCAATGGAGCAAGTCACAGTC | TGCAACATGAATGGATCACCC |
| *Homo* HMGB3 | CCCAGAGGTCCCTGTCAATTT | CGATCATAGCGCACTTTATCTGC |
| *Homo* NARS | CTGGTGTTGCGAGATGGTACA | CCGTGGACAAGAGAACTCCATT |
| *Homo* RFTN1 | ATGGGTTGCGGATTGAACAAG | AGCGGTATTCATAGGACACATCT |
| *Homo* NOS2 | TTCAGTATCACAACCTCAGCAAG | TGGACCTGCAAGTTAAAATCCC |
| *Homo* PRKCB | AGCCCCACGTTTTGTGACC | GCTGGGAACATTCATCACGC |
| *Homo* ARG1 | CCCTGGGGAACACTACATTTTG | GCCAATTCCTAGTCTGTCCACTT |
| *Homo* TGFB1 | GGCCAGATCCTGTCCAAGC | GTGGGTTTCCACCATTAGCAC |
| *Homo* CCL5(1050-1064) | AGCAAGTCACTCCTGCTCAC | ACTGTAAGGCCTTCTTGGGC |
| *Homo* CCL5(1727-1741) | CTTGTTGTCCCCAAGAAAGCG | TCCTCTTTGACCAAGCACCAA |
| *Homo* CCL5(1869-1883) | AGACTCGAATTTCCGGAGGC | CCAAGCATTGGCCGGTATCA |
| *Homo* IRF9(clip1) | TCCTCCAGAGCCAGACTACT | CAATCCAGGCTTTGCACCTG |
| *Homo* IRF9(clip2) | CCATCTCCTGGAATGCACCC | GTCTCTGCAGAAGTAGGCGG |
| *Homo* IRF9(clip3) | TCAGAACCGCCTACTTCTGC | TTACCTGGAACTTCGGTGGG |
| *Homo* IRF9(E6-7-8) | TTACAGACACAACTGAGGCCCC | CTTCACTGTGATAAGATTCTGTGGA |

| **Supplementary Table 3.** Cytokines significantly changed | | | | | | | | | | |
| --- | --- | --- | --- | --- | --- | --- | --- | --- | --- | --- |
| proteinID | AveExp.Y1089 | AveExp.NC | logFC | foldchange | regulation | entrezID | uniprotID | Y1089 | NC | Threshold |
| PDGF-BB | 10.7985695 | 12.1047625 | -1.3061931 | 0.40438655 | down | 5155 | P01127 | 1780.12056 | 4403.5 | TRUE |
| RANTES | 11.4148298 | 12.5353361 | -1.1205063 | 0.45993238 | down | 6352 | P13501 | 2729.27361 | 5935.25 | TRUE |
| M-CSF | 8.76125277 | 9.74651432 | -0.9852615 | 0.50513414 | down | 1435 | P09603 | 432.910225 | 858 | TRUE |
| IP-10 | 7.80062951 | 8.74146699 | -0.9408375 | 0.52093039 | down | 3627 | P02778 | 221.958209 | 427 | TRUE |
| IL-11 | 7.93027835 | 8.45327063 | -0.5229923 | 0.69592692 | down | 3589 | P20809 | 242.922384 | 349.5 | TRUE |
| s TNF RI | 12.7871446 | 13.1889751 | -0.4018305 | 0.75689732 | down | 7132 | P19438 | 7067.28563 | 9337.5 | TRUE |
| IL-6sR | 12.4540456 | 12.75567 | -0.3016244 | 0.81133838 | down | 3570 | P08887 | 5610.01338 | 6914.75 | TRUE |
| IL-10 | 10.1871318 | 9.88493365 | 0.30219817 | 1.23302169 | up | 3586 | P22301 | 1164.822 | 944.5 | TRUE |
| GM-CSF | 10.3590438 | 10.0188956 | 0.34014822 | 1.26588664 | up | 1437 | P04141 | 1312.35739 | 1036.5 | TRUE |
| IL-1a | 10.2204695 | 9.83328544 | 0.38718411 | 1.30783824 | up | 3552 | P01583 | 1192.07543 | 911.25 | TRUE |
| IL-13 | 10.431463 | 10.0337671 | 0.39769586 | 1.3174022 | up | 3596 | P35225 | 1379.96686 | 1047.25 | TRUE |
| MCP-1 | 10.5897267 | 10.1324997 | 0.45722701 | 1.37290044 | up | 6347 | P13500 | 1540.08075 | 1121.5 | TRUE |
| GCSF | 10.0015489 | 9.53138146 | 0.47016744 | 1.38527024 | up | 1440 | P09919 | 1024.09998 | 739 | TRUE |
| TGF-b1 | 10.3987915 | 9.70606427 | 0.69272719 | 1.61633606 | up | 7040 | P01137 | 1349.0447 | 834.25 | TRUE |
